# Identifying polymorphic cis-regulatory variants as risk markers for lung carcinogenesis and chemotherapy responses in tobacco smokers from eastern India

**DOI:** 10.1038/s41598-023-30962-9

**Published:** 2023-03-10

**Authors:** Debmalya Sengupta, Pramiti Mukhopadhyay, Souradeep Banerjee, Kausik Ganguly, Prateek Mascharak, Noyonika Mukherjee, Sangeeta Mitra, Samsiddhi Bhattacharjee, Ritabrata Mitra, Abhijit Sarkar, Tamohan Chaudhuri, Gautam Bhattacharjee, Somsubhra Nath, Susanta Roychoudhury, Mainak Sengupta

**Affiliations:** 1 Department of Genetics, University of Calcutta, Kolkata, West Bengal India; 2 National Institute of Biomedical Genomics, Kalyani, West Bengal India; 3 Department of CHEST, IPGME&R, Kolkata, West Bengal India; 4 Saroj Gupta Cancer Centre and Research Institute, Thakurpukur, West Bengal India; 5 CSIR-Indian Institute of Chemical Biology (TRUE Campus), Kolkata, West Bengal India; 6 Present Address: Department of Cell Systems and Anatomy, University of Texas Health Science Center, San Antonio, Texas USA; 7 Present Address: Department of Biochemistry and Molecular Biology, Indiana University School of Medicine, Indianapolis, Indiana USA; 8 Present Address: Department of Biochemistry and Biophysics, University of Kalyani, Kalyani, West Bengal India; 9 Institute of Health Sciences, Presidency University, Kolkata, West Bengal India

**Keywords:** Cancer, Cancer genetics, Cancer screening, Lung cancer, Cancer genetics, Gene expression, Gene regulation, Genetic association study, Genetic markers, Genotype, Heritable quantitative trait, Population genetics

## Abstract

Aberrant expression of xenobiotic metabolism and DNA repair genes is critical to lung cancer pathogenesis. This study aims to identify the cis-regulatory variants of the genes modulating lung cancer risk among tobacco smokers and altering their chemotherapy responses. From a list of 2984 SNVs, prioritization and functional annotation revealed 22 cis-eQTLs of 14 genes within the gene expression-correlated DNase I hypersensitive sites using lung tissue-specific ENCODE, GTEx, Roadmap Epigenomics, and TCGA datasets. The 22 cis-regulatory variants predictably alter the binding of 44 transcription factors (TFs) expressed in lung tissue. Interestingly, 6 reported lung cancer-associated variants were found in linkage disequilibrium (LD) with 5 prioritized cis-eQTLs from our study. A case–control study with 3 promoter cis-eQTLs (*p* < 0.01) on 101 lung cancer patients and 401 healthy controls from eastern India with confirmed smoking history revealed an association of rs3764821 (*ALDH3B1*) (OR = 2.53, 95% CI = 1.57–4.07, *p* = 0.00014) and rs3748523 (*RAD52*) (OR = 1.69, 95% CI = 1.17–2.47, *p* = 0.006) with lung cancer risk. The effect of different chemotherapy regimens on the overall survival of lung cancer patients to the associated variants showed that the risk alleles of both variants significantly decreased (*p* < 0.05) patient survival.

## Introduction

Exposure to tobacco smoke in active and passive modes is a significant player in the etiology of lung cancer. A high risk of tobacco smoke-induced lung cancer is prevalent in heavy and light smokers^1–4^. However, all individuals exposed to the same type and dose of tobacco smoke do not develop the disease^5^. Epidemiological data reveals that about 15–20% of smokers develop lung cancer while the rest evades the malady^4,5^, suggesting the existence of individual susceptibility. Although microarray analysis and SNP-based association studies have implicated many genes associated with lung carcinogenesis in tobacco smokers, the precise genetic risk signature(s) or prognostic marker(s) is still obscure.

Aberrant expression of xenobiotic metabolism and DNA repair genes is a hallmark of lung cancer^6–9^. The Phase I and Phase II xenobiotic-metabolizing genes (XMGs) are involved in the active clearance of tobacco smoke components that prevents subsequent oxidative stress-induced DNA damage in the pulmonary cells. Some of these genes function in the bio-activation of pro-carcinogenic tobacco smoke components into highly reactive and potent carcinogens resulting in increased carcinogen load in the lung cells^10–12^ causing DNA lesions. The increased burden of carcinogenic metabolites in the pulmonary cells causes increased genomic insults leading to DNA lesions^13–15^. Increased risk of smoking-induced lung cancer is, thus, not only due to exogenous/tobacco smoke contents but their interactions with genes involved in their detoxification or bio-activation^10–12^ and the extent and efficiency of repair of DNA damage^16,17^ caused by tobacco smoke. Microarray and RNA-seq analysis revealed differential expression patterns of XMGs and DNA repair genes (DRGs) in the airway bronchial epithelium of healthy smokers (HS)^18^ compared to healthy non-smokers (HNS)^14,17^ as well as in smokers with lung cancer (SLC) (Supplementary material Fig. S1). Therefore, genes with higher expression in smokers than non-smokers indicate their role in response to tobacco smoke, and their lower expression in lung cancer patients could be due to their inherent ineffective status. However, some genes overexpress in lung cancer patients and increase the carcinogenic load within the cells due to the bioactivation of smoke metabolites.

The regulation of differential gene expression could be due to the variations in the cis-regulatory elements of the gene concerned, often present at long-range upstream or downstream to the transcription start sites. The ENCODE (ENCyclopedia Of DNA Elements)^19,20^ has revealed the genomic positioning of DNase I hypersensitive sites (DHS), which are open chromatin structures accessible to DNA binding proteins like transcription factors^21^. Transcriptional regulation by proximal or distal DHS could be modulated by single nucleotide variants through alteration in the transcription factor (TF)-binding and structural looping^22–24^. Thus, these DHS-SNVs could be responsible for the aberrant expression of XMGs and DRGs in a certain fraction of the smoker population, resulting in the accumulation of carcinogens within the pulmonary cells causing oxidative DNA lesions^17^, which, if not repaired effectively, might lead to a tumorigenic transformation of the cells. These SNVs, individually or together^25^, could act as risk markers of lung cancer, conferring an inherited predisposition in specific individuals.

The standard adjuvant chemotherapy regimens include platinum-based drugs, which are ineffective in increasing the median life expectancy of lung cancer patients and are also extensively toxic^26,27^. Earlier investigations have reported differential gene expression as a predictor for determining patient-specific chemotherapy regimens^28^ and polymorphic variants’ role in modifying chemotherapeutics’ sensitivity and efficacy on different cancers^29,30^. Bioactivation and bioavailability of chemotherapeutic drugs depend on phase I and phase II xenobiotic metabolism enzymes, making them a central player in the efficacy of lung cancer treatment^31^. Moreover, most standard chemotherapy drugs introduce DNA damage, which, if repaired, leads to lesser efficacy of the drugs^32^. Therefore, the differential expression of specific lung cancer-associated genes from xenobiotic metabolism and DNA repair pathways due to cis-regulatory variants could modulate the efficacy of standard adjuvant chemotherapy.

Therefore, this study aims to identify, annotate and prioritize the DHS-SNVs of xenobiotic metabolism and DNA repair genes as genetic susceptibility markers for lung cancer in tobacco smokers, followed by a case–control association study on the eastern Indian population. Further, we aimed to evaluate the role of lung cancer-associated regulatory SNVs on the effect of standard chemotherapy drugs used to treat the patients and their overall survival.

## Materials and methods

### Selection of candidate genes

We followed a detailed literature search to identify xenobiotic metabolism and DNA repair genes showing differential expression between lung cancer and healthy individuals. Following this, we checked the SEGEL database^33^ for expressional differences between HNS and HS groups. We considered all the genes that showed differential expression (*p* < 0.05) and no significant expressional differences between the HNS and HS groups in more than two lung cell types. We did not consider the alveolar macrophage cell type from the SEGEL database in our study. Similarly, we listed median-gene expression of the same set of genes between HS and SLC individuals reported in the literature^4,7,9^ and ONCOMINE^34^ considering fold change ≥ 1.5 and *p* < 0.05. Further, we validated the expression of the selected genes by comparing their expression between the lung adenocarcinoma (LUAD) and/or lung squamous cell carcinoma (LUSC) RNA-seq datasets of The Cancer Genome Atlas (TCGA) and normal lung epithelium from GTEx processed and presented as a web server, GEPIA (Gene Expression Profiling Interactive Analysis)^35^ (http://gepia.cancer-pku.cn/). Based on our hypothesis, we listed those genes that showed differential normalized median expression considering fold change ≥ 1.5 and *p*-value < 0.05 between the LUAD/LUSC and GTEx datasets as our selection criteria for our SLC vs. HS group. Finally, we selected those genes that showed reciprocal expressional patterns between HS vs. HNS and SLC vs. HS groups.

### Selection of candidate DHS and DHS-SNVs

We curated the top 10 expression-correlated DHS (GRCh37/hg19 human genome assembly; cut-off *p* < 0.05) from the "*Regulatory Elements Database*" (http://DNase.genome.duke.edu/)^21,36,37^ for each of the selected genes. According to Sheffield et al.^21^, this method calculates Pearson correlation across samples between gene expression and normalized DNase I scores for each DHS within 100 kb of each gene. A minimum value for DNase I signal and gene expression is set, followed by the calculation of permutation *P*-value using the null distribution of DHS correlations for each gene to a random sample of 10,000 DHSs from different chromosomes (*p* < 0.05). We obtained the SNVs within such selected DHS from the UCSC Table Browser^38^ (http://genome.ucsc.edu/). The *UCSC Table Browser* was configured to our desired settings by changing the default assembly parameter to Feb 2009.GRCh37/hg19″, “*group: variation*”, “*track: common SNPs (141)*”, “*table: All SNPs (141)*”.

### Computational prioritisation of ﻿DHS-SNVs

We used the ENCODE data analyzing tools: rSNPBase 1.0^39^ and RegulomeDB v 1.1^40^ to prioritize DHS-SNVs to ascertain their regulatory potential. We performed SNV enrichment analysis for the rSNPBase and RegulomeDB filtering steps for the DHS-SNVs compared to a universe of randomly selected SNVs. For all the 23 XMGs and 25 DRGs, we selected the transcription start sites (TSS) ± 100 kb region and extracted all the SNVs listed in the dbSNP build 141. Among this pool of SNVs, we randomly selected 1720 SNVs from the XMGs and 1264 SNVs from DRGs as the universe of SNPs. Then, we performed Fisher’s exact text to evaluate the difference in the outcomes between the DHS-SNVs and the universe of SNVs at 5% level of significance. Further, we assessed the impact of DHS-SNVs in genotype-specific transcriptional regulation of target genes in normal healthy post-mortem lung tissue from GTEx Portal v6 (https://www.gtexportal.org/home/)^41–43^. Similarly, as mentioned above, we performed SNV enrichment for the GTEx filtering step for the DHS-SNVs compared to the universe of randomly selected SNVs. Lung cell-type-specific DHS of the genes were obtained from the *Regulatory Elements Database*^21^, considering DHS peak for at least one lung cell type. Further, LD blocks of the cis-eQTLs were obtained from HaploReg v4.1 (https://pubs.broadinstitute.org/mammals/haploreg/haploreg.php)^44^ based on the information from 1000 genome phase 3 data. The gain or loss of transcription factor binding sites (TFBS) due to rSNVs from position weight matrices (PWM) listed in JASPAR^45^ and ENCODE motif libraries were statistically (*p*_impact_ < 0.001) evaluated in an R-based web server, known as *“atSNP”* (http://atsnp.biostat.wisc.edu/)^46^. Further, the expression of such TFs in lung cancer was determined from the *Database of Transcription Factors for Lung Cancer (DbTFLC)* (https://vit.ac.in/files/database/Home.php)^47^.

### Regulatory functional annotation of prioritized rSNVs

Further, we assessed the prioritized rSNVs for more functional attributes that justify their cis-regulatory role in modulating lung cancer risk through the following analyses:

#### Epigenomic signatures at the rSNVs

According to their epigenetic marks, we classified the identified cis-eQTLs (rSNVs) into functional chromatin domains, such as enhancers, promoters, and insulators. The data was obtained from HaploReg v4.1^44^, which hosts the epigenomic data of the Roadmap Epigenomics consortium 2015^48,49^.

#### Cis-eQTLs in lung cancer

The PancanQTL web server (http://bioinfo.life.hust.edu.cn/PancanQTL/)^50^ contains the processed cis-eQTL mapped data on 33 different cancers from The Cancer Genome Atlas (TCGA) raw data. We used this webserver to analyze the cis-eQTL mapping of the prioritized rSNVs of the selected XMGs and DRGs in lung cancer datasets.

#### Linkage disequilibrium (LD) block of rSNVs

We prioritized the LD SNPs (r^2^ ≥ 0.8) of the prioritized rSNVs for their association with lung cancer and other carcinogen-induced cancer. In addition, we obtained the LD block SNPs from HaploReg v4.1^44^ for each of the queried rSNVs, which was taken from the 1000 Genome Project Phase 3 data. Finally, we checked for an indirect association of the rSNVs with lung cancer by itself being in LD with lung cancer-associated SNPs.

#### Co-occurrence of risk alleles and unweighted genetic risk scores

Furthermore, we assessed the co-occurrence of risk alleles of the prioritized rSNVs for all the 26 populations listed in the 1000 Genome^51^ Phase 3 data to identify the risk population based on their unweighted genetic risk scores. In addition, we calculated the unweighted genetic risk score (*uGRS*)^52^ (i.e., the summation of the number of risk alleles across all the prioritized rSNVs) for each 1000 Genome Project enlisted population.

#### Interactome analysis

We performed an interactome analysis for the prioritized protein coding in STRING v10.5 (http://string-db.org)^53,54^, including known and predicted protein–protein interactions. The interactome was expanded to gain more interactors, with a required confidence score > 0.4 as the cut-off.

### Case–control association analysis in a representative population from eastern India

#### Selection of the study subjects

This study included lung cancer patients (*n* = 101) from Saroj Gupta Cancer Centre and Research Institute and the Department of CHEST, IPGME&R in Kolkata. We recruited clinico-radiologically confirmed healthy smokers (n = 401) above 55 years^55^ of age and without any history of cancer as controls. The patients and controls belong to the same geographical region with a confirmed smoking history. We did not consider former smokers (who had quit smoking ≥ 15 years) for the study. First, a detailed questionnaire that included age at sample collection, ethnicity, pack-years, and tumor details like histotype, and TNM staging^56^, were filled up under medical supervision. Then, we noted a detailed account of the followed treatment regimen, including drug combinations, dosages, cycles, responses, and survival time and status. All patients received platinum-based doublet chemotherapy consisting of either cisplatin or carboplatin and another drug in combination. Initially, the patients received 4 cycles of chemotherapy with careful observations of their responses. The treatment was stopped if significant toxicity was observed; otherwise, it was extended to 6 cycles.

#### Collection of blood samples

We collected 10 ml of peripheral blood by venipuncture from lung cancer patients and healthy controls under the supervision of our collaborating clinicians in ethylene-diamine-tetraacetic acid (EDTA) coated tubes. Before sample collection, we obtained informed written consent from the subjects or their family members for voluntary participation in the study.

#### Isolation of genomic DNA and genotyping

We performed the conventional phenol–chloroform method^57^ to isolate genomic DNA and store them at − 20 °C. In addition, we used the PCR–RFLP method for genotyping. Primer sequences were custom designed in Primer3 software (http://bioinfo.ut.ee/primer3-0.4.0/primer3/) and purchased from Integrated DNA Technologies (IDT), USA. The restriction enzymes (New England Biolabs) and their cut patterns were determined from NEBcutter V2.0 (http://nc2.neb.com/NEBcutter2/). We performed the PCR with the reaction mixture (20 µl) containing 50–80 ng of genomic DNA, 20 pmol of each primer, 10 μl of 2X GoTaq PCR Master Mix (Promega), and adjusted the final volume to 20 µl with nuclease-free water. After the quality check, the PCR amplicons were digested with their respective restriction enzymes following the manufacturer’s (NEB) protocol and run on 12% polyacrylamide gels (non-SDS) with 100 bp DNA Ladder (Promega, Cat No. G2101). Three independent individuals verified each gel, 2 without having prior knowledge of the case/control status of the subjects, to avoid biased genotype calls. Further, we confirmed the genotype status of ~ 10% of the study subjects by Sanger Sequencing.

### Statistical analyses

The statistical analyses were performed in R Version 3.4.2^58^, considering statistical significance at *p* < 0.05 (two-sided). We performed a goodness of fit chi-square test to assess the Hardy–Weinberg equilibrium status of the variants in our control population. Student *t*-test and Pearson’s chi-square tests evaluated the association of allele distribution and the demographic variables with lung cancer. Further, we performed logistic regression in additive, dominant, and recessive genetic models to assess the odds ratio (OR), standard error (SE), and 95% confidence intervals (95% CI) adjusted for covariates to measure the association of the rSNVs with lung cancer risk. We also conducted a subgroup analysis and effect modification test for the rSNPs stratified by covariate status on lung cancer risk.

Furthermore, we have independently replicated all the 22 rSNVs from the C34-Malignant neoplasm of bronchus and lung dataset of the UK Biobank hosted in the Gene Atlas webserver (http://geneatlas.roslin.ed.ac.uk/)^59^. We performed a Kaplan–Meier log-rank test that estimated the overall survival (*OS*) distribution for each lung cancer-associated rSNVs. The multivariate Cox-proportional hazard model was used to assess the effect of each rSNPs on the OS of lung cancer patients, adjusted for age, sex, and pack-years of smoking. Finally, we used the Cox hazard methodology to evaluate the relationship between *OS* and the rSNVs stratified by drug combinations of the treatment regimen. The patients that showed complete or partial responses to the treatment were categorized as responders.

In contrast, patients with stable disease, poor responses, or progressive disease were grouped as non-responders. The time to event for the survival analysis was considered a negative outcome, i.e., time to death from the administration of the therapy. The Kaplan–Meier log-rank and Cox-proportional Hazard tests were done in R using *survival*^60^ and *survminer*^61^ packages. Furthermore, we compared the responses of the first and second chemotherapy regimens with the OS of the lung cancer patients considering the effects of the variants rs3764821 (*ALDH3B1*) and rs3748523 (*RAD52*) through a Cox proportional hazards model.

### Ethics approval and consent to participate

The Ethics Committee of Saroj Gupta Cancer Centre and Research Institute (IEC SGCCRI Ref No-2017/MS/1; dated: 11.10.2017), IPGME&R (Memo No. Inst/IEC/2015/545; dated: 10.12.2015), Kolkata and the University of Calcutta (Ref No: 0024/16-117/1434; dated: 24.10.2016), Kolkata, India; approved the study with human subjects as per the regulation of the Indian Council of Medical Research (ICMR) following the Declaration of Helsinki, 1964. Informed consent was obtained from all individual participants included in the study.

## Results

### Gene prioritization

**Figure 1:**
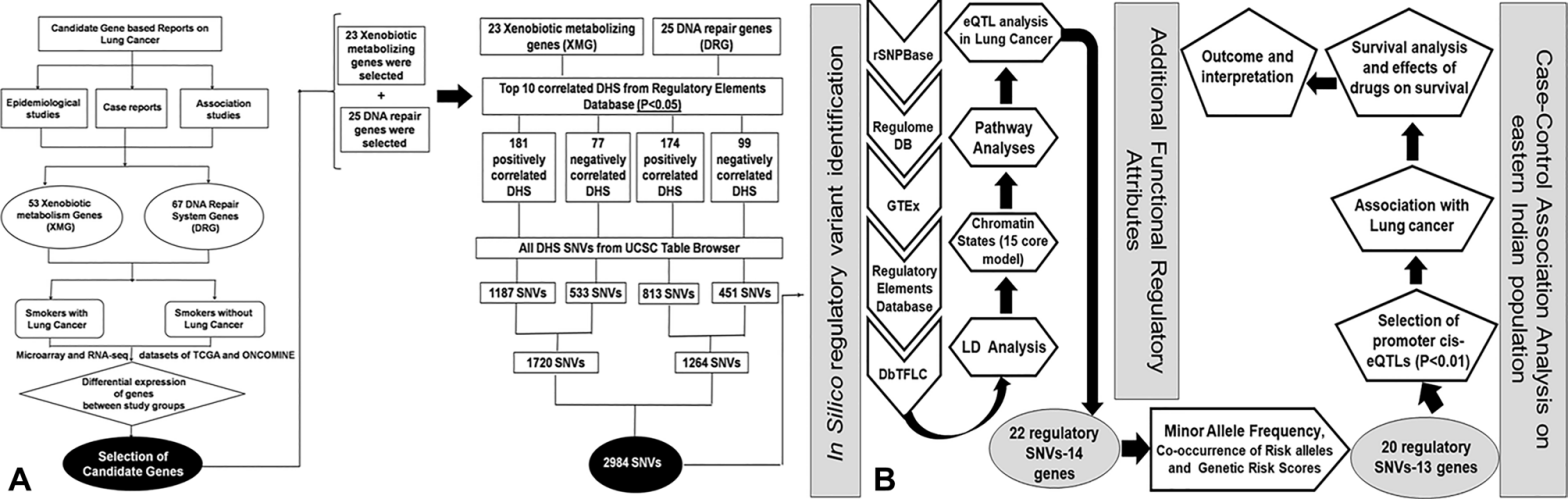
Pathway analysis for identifying regulatory genetic loci as susceptibility markers conferring risk towards tobacco smoke-induced lung carcinogenesis. (**A**) After a detailed literature review on epidemiological reports, association studies, expression studies, and case studies followed by ONCOMINE and TCGA validation, important xenobiotic metabolism and DNA repair genes were selected in cigarette smoke-induced lung cancer. (**B**) The selection and screening of genes, their DNase I hypersensitive sites, and SNVs within the DNase I hypersensitive sites by step-wise use of in silico tools and databases to prioritize potential regulatory SNVs as susceptibility loci in lung carcinogenesis with replications in case–control cohorts. DHS, DNase I hypersensitive sites, eQTL, expression quantitative trait loci, XMG, xenobiotic metabolism gene group, DRG, DNA repair gene group, rSNP, regulatory single nucleotide polymorphism, MAF, minor allele frequency.

Text mining revealed 53 xenobiotic metabolism genes (XMG) and 67 DNA repair genes (DRG) as contenders for identifying rSNVs. These genes are potential candidates for tobacco smoke metabolism and smoke-induced DNA damage repair (Fig. 1). Among the 53 XMG and 67 DRG, further analysis of microarray datasets of ONCOMINE and SEGEL, and RNA-seq dataset of GEPIA, revealed 34 XMG and 17 DRG to be up-regulated, 11 XMG and 26 DRG to be down-regulated, 7 XMG and 24 DRG with no significant difference in the median expression and 3 XMG to be inconclusive when healthy smokers (HS) were compared to healthy non-smokers (HNS). The set of genes as obtained were clustered as ‘*Set A*.’ Again, among the 53 XMG and 67 DRG, 23 XMG and 38 DRG were down-regulated; 23 XMG and 22 DRG as up-regulated; only 4 XMG and 1 DRG were found with no significant expressional change in Smoker Lung Cancer (SLC) group compared to HS. These were grouped as ‘*Set B*’ for the mentioned study groups (Supplementary material Table S1 and Fig. S2). Five XMG and 6 DRG showed inconclusive results. For the xenobiotic metabolism gene set, the selection of genes was segregated into the following categories: (a) genes that were found to be up-regulated in ‘*Set A*’ but down-regulated in ‘*Set B*,’ (b) genes down-regulated in ‘*Set A*’ but up-regulated in ‘*Set B*,’ and, (c) genes with no significant expressional change in ‘*Set A’* but downregulated in ‘*Set B*.’ For DRGs, the genes belonging to category (b) were not considered for further prioritization because the higher expression of DNA repair genes should not render individuals susceptible to tobacco smoke-induced lung carcinogenesis. Our gene prioritization pipeline revealed 23 XMG and 25 DRG potential susceptibility markers for tobacco smoke-induced lung carcinogenesis (Fig. 1). Therefore, 43.4% of total XMGs and 37.31% of total DRGs show significant differential expression between SLC and HS.

### Curation of expression-correlated DHS and DHS-SNVs

From the Regulatory Elements Database, we curated 370 expression-correlated DHS (*p* < 0.05) for the 48 prioritized genes. The regulatory elements database enlists DHS sites, which showed correlations between DNase hypersensitivity to the expression of the nearest genes. For example, we listed 181 positively and 77 negatively correlated DHS coordinates for 23 XMGs. Similarly, 174 positively correlated and 99 negatively correlated DHS coordinates were found for 25 DRG (Supplementary material Table S2). Furthermore, screening for SNVs within these DHS sites revealed 1720 SNVs for xenobiotic metabolism genes, of which 1187 SNVs belonged to positively correlated DHS and 533 SNVs to negatively correlated DHS. Similarly, 1264 SNVs were obtained for DNA repair genes that consist of 813 SNVs within positively correlated DHS and 451 SNVs within negatively correlated DHS (Fig. 1).

### Functional annotation and prioritization of DHS-SNVs

Analysis of 1720 SNVs of xenobiotic metabolism and 1264 SNVs of DNA repair genes in rSNPBase 1.0 revealed 526 SNVs (OR_enrichment_ = 1.48, 95% CI = 1.27–1.72, *p*_enrichment_ = 3.3 × 10^−7^) and 609 SNVs (OR_enrichment_ = 1.37, 95% CI = 1.18–1.59, *p*_enrichment_ = 2.4 × 10^−5^) as ‘*rSNPs*,’ respectively based on various regulatory features such as the proximal and distal regulatory effect of the SNV, RNA binding protein-mediated regulation, and miRNA-mediated regulation in an SNV-specific manner (Supplementary material Table S3). The 1135 SNVs (526 SNVs + 609 SNVs) obtained from rSNPBase were then queried to RegulomeDB v1.1. Scores ranging between 1a to 1f indicate a high regulatory potential of the SNV concerned. Scores between 2a to 3b depict evidence of transcription factor binding disruption without any evidence of QTL; score 4 implies supporting evidence of transcription factor binding and DNase peak. In contrast, scores 5 and 6 depict minimal to no evidence for regulatory annotation of the SNVs^37^. We selected 419 SNVs (OR_enrichment_ = 3.40, 95% CI = 2.86–4.04, *p*_enrichment_ = 2.2 × 10^−16^) from the XMG set and 392 SNVs (OR_enrichment_ = 3.1, 95% CI = 2.57–3.75, *p*_enrichment_ = 2.2 × 10^−16^) from the DRG set with scores between 1a to 4 for further prioritization (Supplementary material Table S4). GTEx portal (http://www.gtexportal.org/home/) revealed 13 SNVs from 7 XMG (OR_enrichment_ = 2.13, 95% CI = 1.03–4.37, *p*_enrichment_ = 0.037) and 9 SNVs from 7 DRG (OR_enrichment_ = 2.64, 95% CI = 1.27–5.87, *p*_enrichment_ = 0.006) as lung tissue-specific cis-eQTLs (*p* < 0.05) (Table 1; Supplementary material Table S5) of the respective genes (Supplementary material Fig. S3). During this analysis, risk alleles were identified based on the genotype-specific expression of the concerned gene following the expressional status in SLC. Ambiguous QTL data that failed to interpret allele-specific expression were not considered for further analysis. The 22 prioritized potential regulatory SNVs (rSNVs) reside within at least one lung cell-type DHS studied in the ENCODE project, justifying tissue-specific transcriptional cis-regulation (Supplementary Material, Table S6). Analysis of these rSNVs through the *atSNP* web server predicts 15 rSNVs to impart statistically significant gain of TFBS for 39 transcription factors (TFs). Similarly, 13 rSNVs were predicted to exhibit a statistically significant loss of TFBS for 28 transcription factors (TFs) (Supplementary material Table S7). Out of these 67 TFs, mining the DbTFLC revealed 44 TFs for 22 rSNVs of 14 genes to express in lung cancer (Supplementary material Table S8). Thus, these 22 rSNVs predictably alter the binding of 44 TFs found to express in lung cancer, further substantiating the loci’s cis-regulatory attribute. HaploReg v4.1 revealed rs1802061C > T (synonymous SNP; Q117Q) and rs4986947G > A (intronic SNP) of *GSTA4* to be in LD. Similarly, rs2153608A > G (intronic SNP) and rs3219472C > T (intronic SNP) of *MUTYH* were also found to be in LD (Supplementary material Table S9).

**Table 1:** Chromatin States and risk allele prediction of DHS-SNVs as cis-eQTLs of the target genes belonging to Xenobiotic metabolism and DNA repair pathway.

Gene symbol	SNP	*p*-value	Effect size	Tissue	Predicted risk allele	cancer type	Beta (β)	t-stat	*p*-value	Risk alleles in lung cancer	The Chromm States in the lung (25-core model)
[I] Xenobiotic metabolism genes											
** SULT1A1**	**rs743590**	**2.00E−11**	**− 0.37**	**Lung**	**G**	**LUAD**	**− 0.2**	**− 4.02**	**6.68E−05**	**G**	**Active Enhancer 1**
SULT1A1	rs3760091	1.40E−12	0.36	Lung	G	No data	No data	No data	No data	No data	Promoter Upstream of TSS
SULT1A1	rs112411210	0.012	0.38	Lung	A	No data	No data	No data	No data	No data	Active Enhancer 2
**GSTA1**	**rs10948723**	**4.00E−07**	**− 0.15**	**Lung**	**C**	**LUAD**	**0.35**	**8.04**	**6.92E−15**	**C**	**Quiescent**
SULT1A2	rs743590	5.50E−06	0.21	Lung	A	No data	No data	No data	No data	No data	Active Enhancer 1
**GSTA1**	**rs2207950**	**6.80E−05**	**0.12**	**Lung**	**A**	**LUAD**	**0.27**	**5.94**	**5.39E−09**	**A**	**Quiescent**
GSTA4	rs1802061	0.0015	− 0.15	Lung	T	No data	No data	No data	No data	No data	Quiescent
ALDH3B1	rs3764821	0.0023	− 0.08	Lung	G	No data	No data	No data	No data	No data	Promoter Downstream of TSS 1
SULT1A2	rs3760091	0.023	− 0.097	Lung	C	No data	No data	No data	No data	No data	Promoter Upstream of TSS
GSTO1	rs12250592	0.027	0.13	Lung	C	No data	No data	No data	No data	No data	Promoter Downstream of TSS 1
GSTO1	rs17883150	0.00085	0.078	Lung	G	No data	No data	No data	No data	No data	Primary DNase site
GSTA4	rs4986947	0.0015	− 0.15	Lung	A	No data	No data	No data	No data	No data	Quiescent
SULT1A2	rs13331376	0.0058	− 0.39	Lung	T	No data	No data	No data	No data	No data	Promoter Upstream of TSS
GSTO1	rs7083465	0.027	0.13	Lung	G	No data	No data	No data	No data	No data	Primary H3K27ac possible Enhancer
MAFG	rs35568625	0.042	− 0.043	Lung	C	No data	No data	No data	No data	No data	Quiescent
[II] DNA Repair genes											
RAD52	rs3748523	2.50E−28	− 0.39	Lung	G	No data	No data	No data	No data	No data	Active TSS
EME2	rs238679	0.00017	− 0.11	Lung	G	No data	No data	No data	No data	No data	Quiescent
EME2	rs1625393	0.0012	− 0.13	Lung	G	No data	No data	No data	No data	No data	Quiescent
ERCC5	rs4150276	0.0009	0.094	Lung	T	No data	No data	No data	No data	No data	Quiescent
MUTYH	rs2153608	0.012	− 0.091	Lung	G	No data	No data	No data	No data	No data	Weak Enhancer 2
MUTYH	rs3219472	0.026	− 0.082	Lung	T	No data	No data	No data	No data	No data	Quiescent
POLM	rs11764344	1.40E−11	− 0.38	Lung	C	No data	No data	No data	No data	No data	Quiescent
** PMS1**	**rs5742926**	**0.00013**	**0.15**	**Lung**	**G**	**LUSC**	**0.29**	**4.11**	**4.72E−05**	**G**	**Promoter Downstream of TSS 1**
MLH1	rs145070498	0.01	− 0.12	Lung	T	No data	No data	No data	No data	No data	Promoter Upstream of TSS

Data from healthy cadaver lung tissue as obtained from the Genotype to Tissue Expression (GTEx) dataset. For lung cancer tissue, cis-eQTL mapping data was obtained from the PancanQTL webserver by analyzing the TCGA data. The chromatin states that data were obtained from HaploRegv4.1 linked to RoadMap Epigenomics, 2015 data.

Normal lung tissue-specific cis-eQTL was calculated, and screening of the rSNVs as cis-eQTL was based on a *p*-value < 0.05*. The risk alleles from the lung cancer group match that of the predicted risk alleles in healthy individuals. FDR corrected *p* < 0.05*. TSS, transcription start site. Lung cancer cis-eQTLs are depicted in bold.

Significant values are in bold.

### Cancer-associated SNPs in LD with the prioritized rSNVs

Text mining of independent candidate association studies revealed 2 prioritized rSNVs, i.e., rs3748523 in the DHS of *RAD52*^62^ and rs4150276 in the DHS of *ERCC5*^63^, reported to be associated with lung cancer previously. The risk allele reported in the literature for these SNVs matches those predicted through our pipeline, thus providing evidence for the precision of our in silico data mining pipeline. We enlisted the 858 LD SNPs (r^2^ > 0.8) for all our 22 predicted rSNVs from HaploReg v4.1 and checked the literature for their association with cancer. Text mining revealed 5 lung cancer-associated SNPs in LD with 5 of our prioritized rSNVs.

Furthermore, 8 SNPs associated with other carcinogen-induced cancers were found in LD, with 6 of our prioritized rSNVs, of which 3 are shared with lung cancer (Supplementary material Table S10). This cross validates 8 of our prioritized rSNVs to be functionally relevant in carcinogenesis. Furthermore, we checked for the association of LD SNPs with lung cancer in the UK Biobank GWAS dataset *C34 Malignant neoplasm of bronchus and lung*, hosted by the Gene Atlas webserver (http://geneatlas.roslin.ed.ac.uk/search/) and found 57 LD-SNPs of 2 prioritized rSNVs associated with lung cancer. Therefore, we obtained 62 (57 + 5) LD-SNPs associated with lung cancer from UK Biobank and literature. Similarly, we curated the 1010 LD-SNPs of randomly selected 22 SNPs from the TSS ± 100 kb region of the 23 XMGs and 25 DRGs. This set of 1010 LD-SNPs was considered the universe of SNPs. Out of these 1010 LD-SNPs, we found 26 LD-SNPs to be associated with lung cancer. Therefore, the LD-SNPs of the rSNVs are significantly enriched (OR_enrichment_ = 2.81, 95% CI = 1.73–4.67, *p*_enrichment_ = 8.78 × 10^−6^). Thus, due to strong LD (r2 > 0.8), there is transitive evidence that the prioritized rSNVs are also associated with lung cancer. In such a case, the prioritized rSNVs could be the causal variants or impart a combinatorial effect on lung cancer pathogenesis with another functional variant. The literature search also revealed 5 coding SNPs from 5 of our prioritized genes associated with lung cancer in Caucasian, Chinese, and Japanese populations (Supplementary material Table S11). This implies a higher risk of tobacco smoke-dependent lung carcinogenesis if the genes harbor the risk alleles of both coding and regulatory polymorphisms leading to significant impairment of gene activity and expression.

We found nominal associations (*p* < 0.05) of three rSNVs, such as rs35568625 (*MAFG*), rs3760091 (*SULT1A2*), and rs743590 (*SULT1A1*), with lung cancer in 1655 cases and 450,609 controls of all white British origin samples from the UK Biobank GWAS dataset *C34 Malignant neoplasm of bronchus and lung*, hosted by the Gene Atlas webserver (http://geneatlas.roslin.ed.ac.uk/search/). However, the three rSNVs, viz. rs3764821 (*ALDH3B1*), rs3748523 (*RAD52*), and rs5742926 (*PMS1*), with which we performed our case–control association study failed to show any association with lung cancer in the *C34 Malignant neoplasm of bronchus and lung* GWAS dataset (Supplementary material Table S12). From the pool of 2984 randomly selected SNPs within the TSS ± 100 kb region of 23 XMG and 25 DRGs, we randomly subsetted 100 SNPs as the universe of SNPs and found only 4 SNPs to be associated with lung cancer in the UK Biobank GWAS dataset *C34 Malignant neoplasm of bronchus and lung* (OR_enrichment_ = 11.05, 95% CI = 1.51–70.31, *p*_enrichment_ = 0.009).

### Epigenomic signatures classifying the rSNVs into chromatin domains

Using the Roadmap Epigenomic data, the 22 prioritized rSNVs were classified by their epigenomic signatures into functional chromatin domains specific to lung tissue. The analysis revealed 4 prioritized rSNVs bearing enhancer marks, 8 rSNVs with transcription start site flanking region/promoter marks, and 11 rSNVs with insulators/ heterochromatin/ repressed region-specific epigenomic marks (Supplementary material Table S13).

### Population segregation based on unweighted genetic risk score

The 1000 Genome data revealed 12 rSNVs from the 9 prioritized XMGs and 8 rSNVs from 8 DRGs to be polymorphic with global MAF > 0.01 (Supplementary Material, Table S14). The mean *uGRS* estimate for each of the geographical populations of the 1000 Genome project for 22 prioritized rSNVs revealed that the Europeans (*uGRS* = 83.51) are at the highest risk of developing tobacco-related lung cancer, followed by the East Asians (*uGRS* = 82.9) and South Asians (*uGRS* = 80.58). The Latin Americans (*uGRS* = 74.71) were at least risk, followed by the Africans (*uGRS* = 79.1) for tobacco smoke-induced lung carcinogenesis. However, the mean *uGRS* calculated for each subpopulation of the 1000 Genome data revealed the Gambians in the Western divisions in the Gambia (GWD) (*uGRS* = 95.92) to be at the highest risk of developing tobacco smoke-induced lung cancer, followed by Yorubans in Ibadan, Nigeria^64^ (*uGRS* = 90.96) and Iberians in Spain (IBS) (*uGRS* = 88.71). On the other hand, Americans of African Ancestry in South West USA (ASW) (*uGRS* = 50.42) are the population at least risk of tobacco smoke-induced lung cancer, followed by people of Mexican Ancestry from Los Angeles, USA (MXL) (*uGRS* = 56.04) and Mende people in Sierra Leone (MSL) (*uGRS* = 69.71). (Supplementary material Table S15).

### Interactome analysis for more candidate genes

The interaction network analysis between the prioritized genes and expanded to 50 more interactors revealed strong associations among the GST family (GSTA1, GSTA4, GSTO1) proteins with a high mean score greater than 0.9. Furthermore, other candidate players, such as TP53, NFE2L2, TPT1, and NF2, involved in apoptosis, cytoskeletal remodeling, cell cycle regulation, cancer stemness, and many critical cancer regulatory pathways, were found to interact with our prioritized protein-coding genes (Supplementary material Fig. S4). The analysis revealed TP53 as the nodal gene that connects the xenobiotic metabolism pathway with apoptosis, DNA repair, cytoskeletal remodeling, and cancer stemness. Furthermore, co-expression of our prioritized genes with other reported lung cancer-associated genes was found, which depicts their possible functional interplay in the disease pathogenesis (Supplementary material Table S16). Pathway analysis revealed a cross-regulation between cytoskeletal remodeling, metastasis, apoptosis, xenobiotic metabolism, DNA repair, and cell cycle regulatory pathways. Such cross-regulation among the pathways reveals the gene regulatory interactome in lung cancer pathogenesis (Supplementary material Tables S17–S20).

### Analysis of mapped cis-eQTL in lung cancer cases

The prioritized rSNVs were further assessed for their cis-regulatory potential in lung cancer cases on the processed TCGA data hosted by the PancanQTL webserver. The analysis revealed a subset of 4 rSNVs as significant cis-eQTLs in both Lung Adenocarcinoma (LUAD) and Lung Squamous cell carcinoma (LUSC) datasets after false discovery rate (FDR) correction (*p*_*FDR*_ < 0.05) (Supplementary material Fig. S5). Furthermore, the risk alleles of these 4 cis-eQTLs match our prediction, indicating the precision and accuracy of the predictive analysis (Table 1).

### Case–control association analysis

#### The clinical and demographic attributes of the study subjects

The study involved 101 smoker cases and 401 smoker controls with a mean age of 58.93 ± 12.29 and 66.18 ± 7.85, respectively, collected from two hospitals in Kolkata. The formula for estimating pack-years of smoking: [(No. of cigarettes/beedis /cigars)/20) × No. of years smoked] showed no significant difference between cases and controls. However, the distribution of males over females is higher in both cases and controls. This contributed to a sex bias in our sampling of controls, for which we were unable to consider the parameter of gender in our association study. The histological subtype Adenocarcinoma (ADC) was found to be the most abundant type of lung cancer, followed by Squamous cell carcinoma (SqCC) and Small cell lung cancer (SCLC).

Furthermore, TNM staging data, available for 99 patients, showed that Stage III and Stage IV were highly over-represented compared to Stage I and Stage II, probably due to late reporting of the patients to oncologists. For 2 patients, TNM staging was not done till the date of sample collection. Nearly 90% of the lung cancer cases of our sample population exhibit distant metastasis (M1), while the remaining patients did not show any sign of metastasis till the date of collection. The clinical and demographic characteristics, including age, sex, pack-years, tumor histology, TNM staging, and metastases, are summarised in (Table 2, Supplementary material Table S21).

**Table 2:** Clinical and demographic characteristics of lung cancer patients and controls.

Variable	Cases, N = 101	Controls, N = 401	*p*-value
Age	–	–	–
< 39	8	0	
40–49	8	0	
50–59	28	72	
60–69	43	212	
≥ 70	14	115	
Mean ± SD	58.65 ± 12.12	66.14 ± 7.83	< 0.001***
Pack years			
< 20	9	103	
20–49	26	156	
≥ 50	66	130	
Mean ± SD	66.92 ± 34.95	59.29 ± 37.47	0.064
Gender			
Male	80	400	< 0.001***
Female	21	1	
Tumour histology			
Adenocarcinoma (ADC)	50		
Squamous cell carcinoma (SqCC)	39		
Small cell lung cancer (SCLC)	13		
Others	1		
TNM staging			
I	2		
II	11		
III	48		
IV	40		
Unknown	2		
Metastasis			
No	9		
Yes	91		

*SD* standard deviation, *N* total number of case-patients or control subjects.

*p*-values for sex were derived from the Chi-square test; the Student t-test was used for age and pack-years. All *P*-values are two-sided. *p* < 0.05 was considered statistically significant.

#### Regulatory polymorphic variants and their association with lung cancer risk

**Figure 2:**
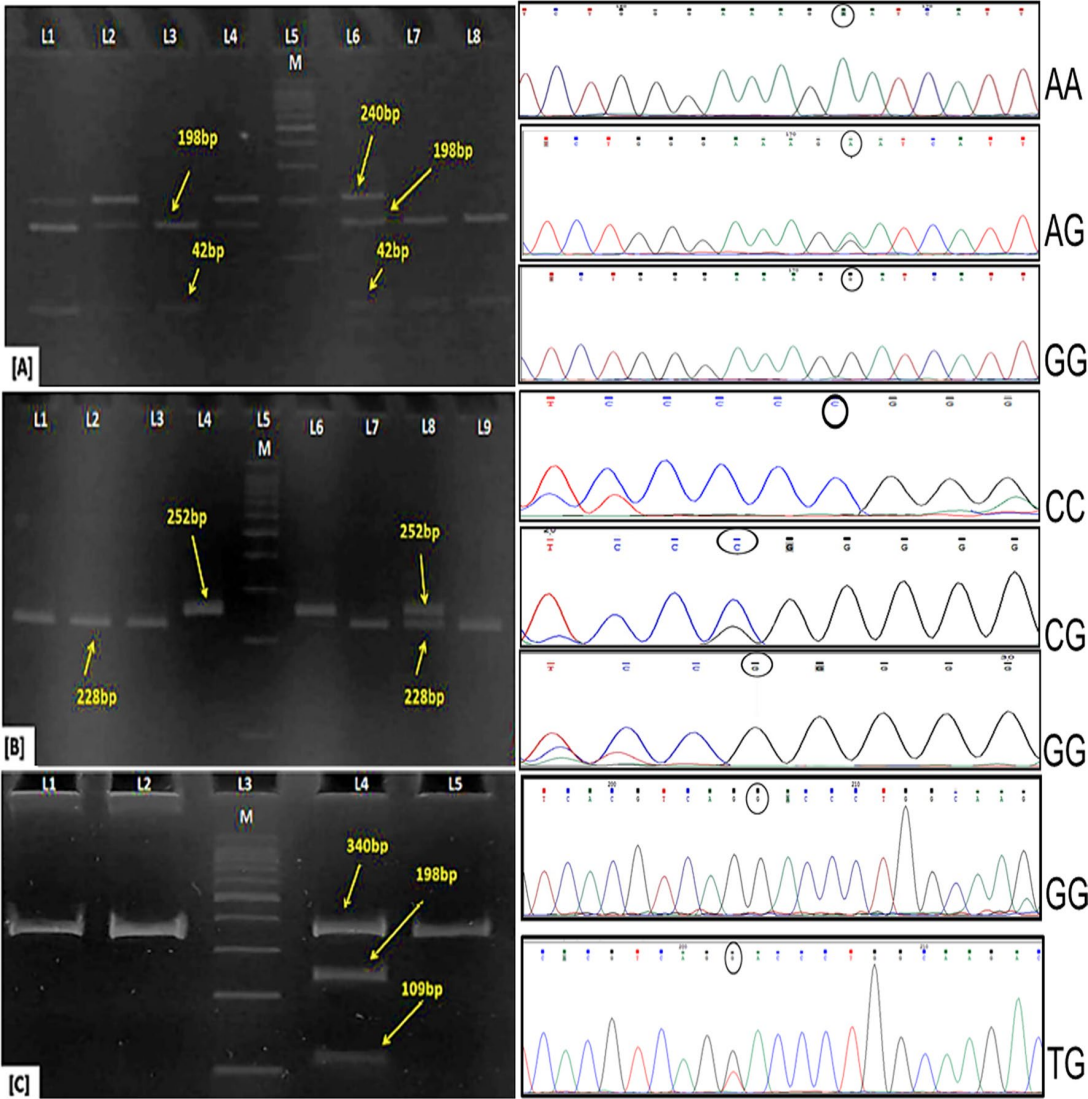
Genotyping of (**A**) rs3764821 of *ALDH3B1*, (**B**) rs3748523 of *RAD52* and (**C**) rs5742926 of *PMS1* by PCR–RFLP method with a representative chromatogram of Sanger sequencing for each genotype of the rSNPs. For rs3764821, Gel 1: Lane 1,2,4,6 depicts AG genotypes with cut patterns as 240 bp, 198 bp, 42 bp; Lane 3, 7, 8 depicts GG genotypes with 198 bp and 42 bp fragments. For rs3748523, Gel 2: Lane 1,2,3,7 & 8 depicts CC genotypes with 228 bp and 24 bp (not visible) fragments; Lane 4 & 6 depicts GG genotypes as uncut (252 bp) fragments; Lane 8 depicts CG genotype with 252 bp, 228 bp and 24 bp fragments. For rs5742926, Gel 3: Lane 1, 2 & 5 depicts GG genotypes as uncut (340 bp) fragments and Lane 4 depicts GT genotype with 340 bp, 231 bp, 109 bp. A representative chromatogram for heterozygous peak is also provided for each rSNP.

The 1000 Genome data revealed 12 rSNVs from the 9 prioritized XMGs and 8 rSNVs from 8 DRGs to be polymorphic with global MAF > 0.01, and we would designate them as SNPs from now on in the text as per the definition of the term (Supplementary Material, Table S21). Out of these 20 rSNPs, 3 promoter rSNPs with GTEx *p*-value < 0.01, i.e., rs3764821 for *ALDH3B1*, rs3748523 for *RAD52,* and rs5742926 for *PMS1,* were selected for our case–control association analysis on the East Indian population. After genotyping, the three rSNPs were found in Hardy–Weinberg equilibrium (Supplementary Material, Table S22). Sanger Sequencing reconfirmed that the genotype calls in about 10% of the total samples. The representative gel and chromatogram pictures are shown in (Fig. 2).

Analysis by Pearson’s chi-square revealed an association between the predicted risk allele of rs3764821-*ALDH3B1* (**G:** OR = 2.54, 95% CI = 1.55–4.15, *p* = 0.00022***) and rs3748523-*RAD52* (**G:** OR = 1.65, 95% CI = 1.13–2.41, *p* = 0.01*) with lung cancer while, no significant association of rs5742926 (*PMS1*) with lung cancer in smokers was found (Table 3).

**Table 3:** Association of 3 promoter cis-eQTLs belonging to Xenobiotic metabolism and DNA repair pathway.

Gene-polymorphism	Genotypes/alleles	Smoker lung cancer cases; n = 101 (%)	Healthy smoker controls; n = 401 (%)	Model	Comparisons	OR (95% CI)^a^	*p*-value^a^	Adjusted OR (95% CI)^b^	*p*-value^b^
*ALDH3B1-rs3764821 A* > *G*	AA	71 (70.3)	345 (86)	Additive	AA vs. AG vs. GG	2.64 (1.63–4.29)	0.00009***	2.51 (1.42–4.67)	0.002**
	AG	28 (27.7)	53 (13.2)	Dominant	(AG + GG) vs. AA	2.69 (1.61–4.50)	0.0002***	2.49 (1.35–4.59)	0.003**
	GG	2 (1.9)	1 (0.3)	Recessive	GG vs. (AA + AG)	8.04 (0.72–89.57)	*0.09*	13.98 (0.85–228.81)	*0.06*
	**Alleles**				**Alleles**				
	A	170 (84.2)	743 (92.6)		A	–			
	G	32 (15.8)	55 (6.9)		**G**	2.54 (1.55–4.15)	0.00022***	–	–
*RAD52-rs3748523 C* > *G*	CC	54 (53.5)	269 (67.1)	Additive	CC vs. CG vs. GG	1.69 (1.17–2.47)	0.006**	1.83 (1.15–2.92)	0.016*
	CG	41 (40.6)	122 (30.4)	Dominant	(CG + GG) vs. CC	1.77 (1.14–2.76)	0.01*	1.73 (1.03–2.92)	0.04*
	GG	6 (5.9)	10 (2.5)	Recessive	GG vs. (CC + CG)	2.47 (0.88–6.96)	*0.09*	5.39 (1.35–21.54)	0.02*
	**Alleles**				**Alleles**				
	C	149 (73.8)	660 (82.3)		C	–			
	G	53 (26.2)	142 (17.7)		**G**	1.65 (1.13–2.41)	0.01*	–	–
*PMS1-rs5742926 G* > *T*	GG	94 (93.1)	346 (86.3)	Additive	GG vs. GT	0.51 (0.22–1.15)	*0.10*	0.52 (0.21–1.31)	*0.17*
	GT	7 (6.9)	51 (12.7)	Dominant	*–*	*–*	*–*	*–*	*–*
	TT	0 (0)	0 (0)	Recessive	*–*	*–*	*–*	*–*	*–*
	**Alleles**				**Alleles**				
	G	195 (96.5)	743 (92.65)		T	–			
	T	07 (3.5)	51 (6.4)		**G**	1.91 (0.82–4.69)	*0.13*	–	–

Pearson’s chi-square test was done to determine allelic association with lung cancer, and multivariate logistic regression was done in additive, dominant and recessive models to ascertain genotypic association with lung cancer. ^a^Unadjusted association with crude odds ratio and 95% confidence interval and p-value. ^b^Adjusted for age, sex, pack-years, alcohol consumption, tobacco chewing, betel quid chewing, wood smoke, coal smoke, asbestos, and pesticide exposures; CI: Confidence interval, OR: Odds ratio, Significance levels: *p* < 0.001 ‘***,’ 0.01 ‘**,’ 0.05 ‘*.’ n = number of cases and controls.

Significant values are in bold and italics.

Unadjusted logistic regression revealed strong association of rs3764821 of *ALDH3B1* (**G** vs. **A**: OR = 2.64, 95% CI = 1.63–4.29, *p* = 0.00009***) and rs3748523 of *RAD52* (**G** vs. **C**: OR = 1.69, 95% CI = 1.17–2.47, *p* = 0.006**) with lung cancer in additive model. In the dominant model, association with lung cancer was found for both rs3764821 (**AG + GG** vs. **AA**: OR = 2.69, 95% CI = 1.61–4.50, *p* = 0.0002***) and rs3748523 (**CG + GG** vs. **CC**: OR = 1.77, 95% CI = 1.14–2.76, *p* = 0.01*). The rSNP, rs5742926 of *PMS1,* has no association with lung cancer (Table 3).

Further, covariate-adjusted logistic regression revealed an association of rs3764821 of *ALDH3B1* in both additive (**G** vs. **A**: OR = 2.51, 95% CI = 1.42–4.67, *p* = 0.002**) and dominant (**AG + GG** vs. **AA**: OR = 2.49, 95% CI = 1.35–4.59, *p* = 0.003**) models. The rSNP, rs3748523 of *RAD52* also revealed a significant association with lung cancer in additive (**G** vs. **C**: OR = 1.83, 95% CI = 1.15–2.92, *p* = 0.016*), dominant (**CG + GG** vs. **CC**: OR = 1.73, 95% CI = 1.03–2.92, *p* = 0.04*) and recessive (**GG** vs. **CC + CG**: OR = 5.39, 95% CI = 1.35–21.54, *p* = 0.02*) effect models (Table 3).

#### Association of the polymorphic regulatory variants with clinicopathological features of lung cancer

We found a significant association of rs3764821 with adenocarcinoma (OR = 2.79, 95% CI = 1.03–7.53, *p* = *0.043*) and SCLC (OR = 5.95, 95% CI = 1.65–21.47, *p* = *0.007*) adjusted for age, sex, and pack-years of smoking. Similarly, rs3748523 was associated with squamous cell carcinoma (OR = 2.32, 95% CI = 1.01–5.34, *p* = *0.046*) adjusted for age, sex, and pack-years of smoking. The Association of the variants with different TNM stages and distant metastases in additive and dominant models is summarized in the supplemental material (Supplementary Material, Table S23).

#### Effect of tobacco smoking on the association of the polymorphic regulatory variants with lung cancer

The sub-group analysis stratified by pack-years revealed a significant association of rs3764821 of *ALDH3B1* in both low pack-years (< 47 py) (OR = 2.58, 95% CI = 1.13–5.88, *p* = 0.024*) and high pack-years (≥ 47 mean py) (OR = 2.73, 95% CI = 1.49–5.01, *p* = 0.0012**) subgroups with risk of lung cancer in the additive model. The rSNP, rs3748523 of *RAD52,* showed significant association only in low pack-years (< 47 mean py) (OR: 1.92, 95% CI = 1.20–3.06, *p* = 0.0062*) subgroup in the additive model. None of the rSNPs was found to show any significant (*p* < 0.05) effect modification on lung cancer risk based on smoking (Supplementary Material, Table S23). None of the other covariates revealed any significant effect on the association of the polymorphic variants with lung cancer (Supplementary Material, Table S24).

#### The combined effect of the polymorphic regulatory variants on lung cancer risk

The association between lung cancer and possible combinations of rs3764821 and rs3748523 was assessed by genotype-genotype combination analysis. Interestingly, we found a significant association between the heterozygous genotypes of rs3764821 and rs3748523 (AG + CG: OR = 2.79, 95% CI = 1.14–6.47, *p* = 0.013) with lung cancer risk (Supplementary Material, Table S25).

#### Association of the polymorphic regulatory variants and the overall survival (OS) of lung cancer patients

We performed a survival analysis for 96 lung cancer patients (Table 4) and assessed the association between overall survival (OS) and the variants rs3764821 and rs3748523, using a univariate analysis expressed in Kaplan–Meier (KM) plots and log-rank test. In addition, we followed a multivariate Cox regression model to adjust various covariates like age, sex, pack-years of smoking, histological subtypes, and TNM stage (Table 4). In this subset of lung cancer patients, the genotypic distribution of rs3764821 (χ2 = 0.24; df = 2; *p* = 0.89) and rs3748523 (χ2 = 0.47; df = 2; *p* = 0.79) was in HWE.

**Table 4:** Relationship of the regulatory polymorphisms with the overall survival (OS) of lung cancer patients, its subtypes, and TNM stages.

Regulatory polymorphisms	Genotypes	Dead	Alive	Median OS (Months)	Crude HR (95% CI)	Log-rank *p*-value*	Adjusted HR^#^ (95% CI)	*p-*value*
[A] Overall Lung Cancer								
* ALDH3B1-rs3764821A* > *G*	AA	36	31	24	1 (Reference)			
	AG + GG	19	10	9	2.07 (1.13–3.79)	0.02*	2.12 (1.16–3.89)	0.015*
* RAD52-rs3748523C* > *G*	CC	25	31	24	1 (Reference)			
	CG + GG	29	11	7	2.19 (1.25–3.87)	0.004**	2.32 (1.30–4.12)	0.004**
[B] Adenocarcinoma								
* ALDH3B1-rs3764821A* > *G*	AA	18	14	24	1 (Reference)			
	AG + GG	10	6	8.9	2.18 (0.93–5.15)	*0.074*	2.35 (0.99–5.59)	*0.053*
* RAD52-rs3748523C* > *G*	CC	15	14	24	1 (Reference)			
	CG + GG	13	6	12	1.47 (0.67–3.21)	*0.33*	1.48 (0.68–3.22)	*0.33*
[C] Squamous Carcinoma								
* ALDH3B1-rs3764821A* > *G*	AA	16	16	24	1 (Reference)			
	AG + GG	4	3	8	1.33 (0.43–4.16)	0.6	1.25 (0.39–3.97)	0.71
* RAD52-rs3748523C* > *G*	CC	6	14	24	1 (Reference)			
	CG + GG	14	2	6	5.25 (1.69–16.21)	0.001**	5.64 (1.76–18.1)	0.003**
[D] SCLC								
* ALDH3B1-rs3764821A* > *G*	AA	1	3	27	1 (Reference)			
	AG + GG	5	2	9	The sample size is insufficient			
* RAD52-rs3748523C* > *G*	CC	4	3	11	1 (Reference)			
	CG + GG	2	3	9	0.94 (0.15–5.66)	*0.94*	1.10 (0.17–7.17)	*0.92*
[E] Stage I + II (Early Stages)								
* ALDH3B1-rs3764821A* > *G*	AA	3	8	7	1 (Reference)			
	AG + GG	1	3	4.5	10.06 (1.04–96.66)	0.01*	2.78 (0.08–99,500,000)	*0.14*
* RAD52-rs3748523C* > *G*	CC	2	8	12	1 (Reference)			
	CG + GG	4	1	6.5	5.73 (0.58–56.38)	*0.09*	219.87 (0.19–246,200)	*0.83*
[F] Stage III + IV (Late Stages)								
* ALDH3B1-rs3764821A* > *G*	AA	32	23	13	1 (Reference)			
	AG + GG	16	10	11	1.64 (0.84–3.18)	*0.1*	1.65 (0.84–3.85)	*0.14*
* RAD52-rs3748523C* > *G*	CC	23	23	9	1 (Reference)			
	CG + GG	24	10	7	2.07 (1.13–3.79)	0.02*	2.32 (1.24–4.31)	0.008**

*p*-value < 0.05*, 0.01**, 0.001***. Hazard ratios, 95% CI, and their corresponding *p*-values were calculated by Kaplan–Meier survival analysis after adjusting for remission and survival in months, and ^#^adjusted hazard ratios, 95% CIs and their corresponding p-values were calculated by Cox regression models adjusted for age, sex, and pack-years of smoking.

Significant values are in bold and italics.

**Figure 3:**
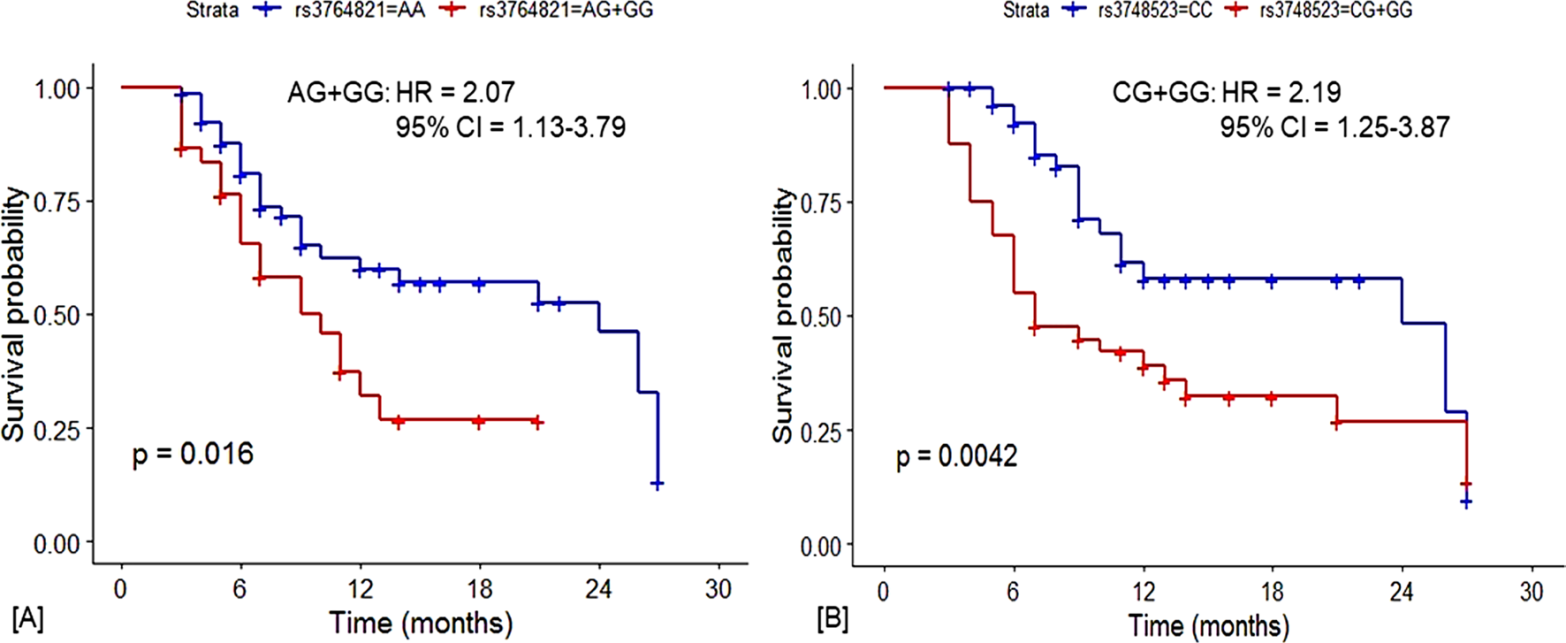
Kaplan–Meier curves depict the association between overall survival in lung cancer patients and the cis-regulatory polymorphic variants in the eastern Indian population. It shows significantly lower overall survival in lung cancer patients with (**A**) rs3764821 (*ALDH3B1*); a combination of heterozygous and homozygous variant genotypes (*AG* + *GG*), and (**B**) rs3748523 (*RAD52*); the combination of heterozygous and homozygous variant genotypes (*CG* + *GG*). Significance at log-rank *p* < 0.05*.

Individuals with the combined heterozygous and homozygous risk genotypes of both variants have a median survival time (MST) of 7 months compared to 9 months for the wild-type genotypes. We found a significant association of rs3764821 (AA vs. AG + GG: hazard ratio [HR] = 2.07; 95% CI = 1.13–3.79; log-rank *p* = 0.02) and rs3748523 (CC vs. CG + GG: hazard ratio [HR] = 2.19; 95% CI = 1.25–3.87; log-rank *p* = 0.004) (Fig. 3; Table 4) with the low OS of lung cancer patients using KM survival analysis and univariate Cox regression model. Multivariate Cox regression analysis revealed a lower *OS* in lung cancer patients for rs3764821 (AA vs. AG + GG: HR = 2.12; 95% C.I. = 1.16–3.89; *p* = 0.015) and rs3748523 (CC vs. CG + GG: hazard ratio [HR] = 2.32; 95% CI = 1.30–4.12; *p* = 0.004) adjusted for age, sex and pack year of smoking (Table 4).

The effect of the rSNPs on the OS of patients with a specific subtype of lung cancer was also evaluated by a multivariate Cox regression model adjusted for age, sex, and pack-years of smoking. The variant rs3748523 was significantly associated with a lower OS of patients with squamous cell carcinoma (N = 36; CC vs. CG + GG: hazard ratio [HR] = 5.64; 95% CI = 1.76–18.1; *p* = 0.003) adjusted for age, sex, and pack-years of smoking. No significant association was observed for the other two lung cancer histological subtypes. The variant rs3748523 (*RAD52*) was found to be significantly associated with lower *OS* (CC vs. CG + GG: hazard ratio [HR] = 2.32; 95% CI = 1.24–4.31; *p* = 0.008) in lung cancer patients of later stages (stage III and stage IV) adjusted for age, sex and pack-years of smoking (Table 4).

#### Effect of polymorphic regulatory variants on the overall survival of lung cancer patients stratified by chemotherapy regimens with different drug combinations

The association of the polymorphic regulatory variants rs3764821 and rs3748523 with the OS of lung cancer patients stratified by chemotherapy regimens with different drug combinations in the dominant model is summarized in a table (Table 5). For some non-responders to the first chemotherapy regimen, treatment was extended up to three chemotherapy regimens with different combinations of drugs.

**Table 5:** Association of regulatory polymorphisms and overall survival according to the chemotherapy regimen.

Regulatory polymorphisms	Genotypes	Crude HR (95% CI)	Log-rank* p*	Adjusted HR^†^ (95% CI)	*p*-value^†^
Regimen 1—Docetaxel cis/carboplatin					
* ALDH3B1-rs3764821A* > *G*	AA	1 (Reference)			
	AG + GG	2.51 (0.35–18.17)	*0.3*	26.29 (0.18–3762.41)	*0.19*
* RAD52-rs3748523C* > *G*	CC	1 (Reference)			
	CG + GG	0.47 (0.04–5.16)	*0.5*	1.55 (0.03–90.37)	*0.83*
Regimen 1—Pemetrexed cis/carboplatin					
* ALDH3B1-rs3764821A* > *G*	AA	1 (Reference)			
	AG + GG	1.28 (0.39–4.15)	*0.7*	1.8 (0.39–8.19)	*0.49*
* RAD52-rs3748523C* > *G*	CC	1 (Reference)			
	CG + GG	2.42 (0.78–7.47)	*0.1*	1.33 (0.29–5.89)	*0.71*
Regimen 1– Paclitaxel cis/carboplatin					
* ALDH3B1-rs3764821A* > *G*	AA	1 (Reference)			
	AG + GG	2.67 (0.93–8.39)	*0.06*	3.62 (1.03–12.71)	0.044*
* RAD52-rs3748523C* > *G*	CC	1 (Reference)			
	CG + GG	3.19 (1.10–9.27)	0.02*	1.95 (0.58–6.58)	*0.28*
Regimen 1– Nanopaclitaxel cis/carboplatin					
* ALDH3B1-rs3764821A* > *G*	AA	1 (Reference)			
	AG + GG	1.15 (0.19–6.98)	*0.9*	3.37 (0.24–47.98)	*0.37*
* RAD52-rs3748523C* > *G*	CC	1 (Reference)			
	CG + GG	7.95 (0.89–71.16)	0.03*	11.32 (0.18–698.64)	*0.25*
Regimen 1—Etoposide cis/carboplatin					
* ALDH3B1-rs3764821A* > *G*	AA	1 (Reference)			
	AG + GG	The sample size is insufficient			
* RAD52-rs3748523C* > *G*	CC	1 (Reference)			
	CG + GG	1.01 (0.20–5.08)	*0.99*	0.78 (0.10–5.97)	*0.82*
Combination Drug Regimen: Docetaxel cis/carboplatin (2nd) * Nanopaclitaxel cis/carboplatin (1st)					
* ALDH3B1-rs3764821A* > *G*	AA	1 (Reference)			
	AG + GG	1.15 (0.19–6.98)	*0.9*	2.05 (0.25–16.55)	*0.5*
* RAD52-rs3748523C* > *G*	CC	1 (Reference)			
	CG + GG	7.15 (0.74–69.03)	*0.05*	16.75 (0.38–734.18)	*0.14*
Combination Drug Regimen: Nanopaclitaxel cis/carboplatin (2nd) * Pemetrexed cis/carboplatin (1st)					
* ALDH3B1-rs3764821A* > *G*	AA	1 (Reference)			
	AG + GG	1.40 (0.38–5.10)	*0.6*	1.39 (0.39–5.31)	*0.62*
* RAD52-rs3748523C* > *G*	CC	1 (Reference)			
	CG + GG	2.27 (0.75–6.84)	*0.1*	2.35 (0.75–7.37)	*0.14*
Combination Drug Regimen: Pemetrexed cis/carboplatin (2nd) * Paclitaxel cis/carboplatin (1st)					
* ALDH3B1-rs3764821A* > *G*	AA	1 (Reference)			
	AG + GG	2.47 (0.78–7.84)	*0.1*	3.25 (0.88–12.06)	*0.08*
* RAD52-rs3748523C* > *G*	CC	1 (Reference)			
	CG + GG	2.97 (1.01–8.76)	*0.05*	1.75 (0.51–6.07)	*0.38*
Combination Drug Regimen: Paclitaxel cis/carboplatin (2nd) * Pemetrexed cis/carboplatin (1st)					
* ALDH3B1-rs3764821A* > *G*	AA	1 (Reference)			
	AG + GG	1.18 (0.32–4.28)	*0.8*	1.21 (0.33–4.48)	*0.78*
* RAD52-rs3748523C* > *G*	CC	1 (Reference)			
	CG + GG	3.10 (0.95–10.16)	*0.05*	3.41 (0.99–11.81)	*0.05*
Combination Drug Regimen: Gemcitabine cis/carboplatin (2nd) * Paclitaxel cis/carboplatin (1st)					
* ALDH3B1-rs3764821A* > *G*	AA	1 (Reference)			
	AG + GG	3.02 (1.09–8.39)	0.03*	4.16 (1.34–12.89)	0.014*
* RAD52-rs3748523C* > *G*	CC	1 (Reference)			
	CG + GG	2.79 (1.09–7.15)	0.03*	2.12 (0.74–6.11)	*0.16*
Combination Drug Regimen: Gemcitabine cis/carboplatin (2nd) * Pemetrexed cis/carboplatin (1st)					
* ALDH3B1-rs3764821A* > *G*	AA	1 (Reference)			
	AG + GG	1.89 (0.74–4.85)	*0.2*	1.86 (0.70–4.91)	*0.21*
* RAD52-rs3748523C* > *G*	CC	1 (Reference)			
	CG + GG	2.21 (0.88–5.51)	*0.08*	2.50 (0.94–6.68)	*0.07*
Combination Drug Regimen: Gemcitabine cis/carboplatin (2nd) * Nanopaclitaxel cis/carboplatin (1st)					
* ALDH3B1-rs3764821A* > *G*	AA	1 (Reference)			
	AG + GG	3.03 (0.78–11.8)	*0.09*	3.63 (0.93–14.08)	*0.06*
* RAD52-rs3748523C* > *G*	CC	1 (Reference)			
	CG + GG	3.28 (0.92–11.67)	*0.05*	3.38 (0.51–22.24)	*0.21*
Combination Drug Regimen: Eribulin cis/carboplatin (3rd) * Nanopaclitaxel cis/carboplatin (2nd) * Pemetrexed cis/carboplatin (1st)					
* ALDH3B1-rs3764821A* > *G*	AA	1 (Reference)			
	AG + GG	1.40 (0.38–5.10)	*0.6*	1.39 (0.37–5.31)	*0.62*
* RAD52-rs3748523C* > *G*	CC	1 (Reference)			
	CG + GG	2.26 (0.75–6.84)	*0.1*	2.35 (0.75–7.37)	*0.14*

*p*-value < 0.05*, 0.01**, 0.001***. Hazard ratios, 95% CI, and their corresponding *p*-values were calculated by Kaplan–Meier survival analysis after adjusting for remission and survival in months, and ^#^adjusted hazard ratios, 95% CIs and their corresponding *p*-values were calculated by Cox regression models adjusted for age, sex, and pack-years of smoking.

Significant values are in bold and italics.

**Figure 4:**
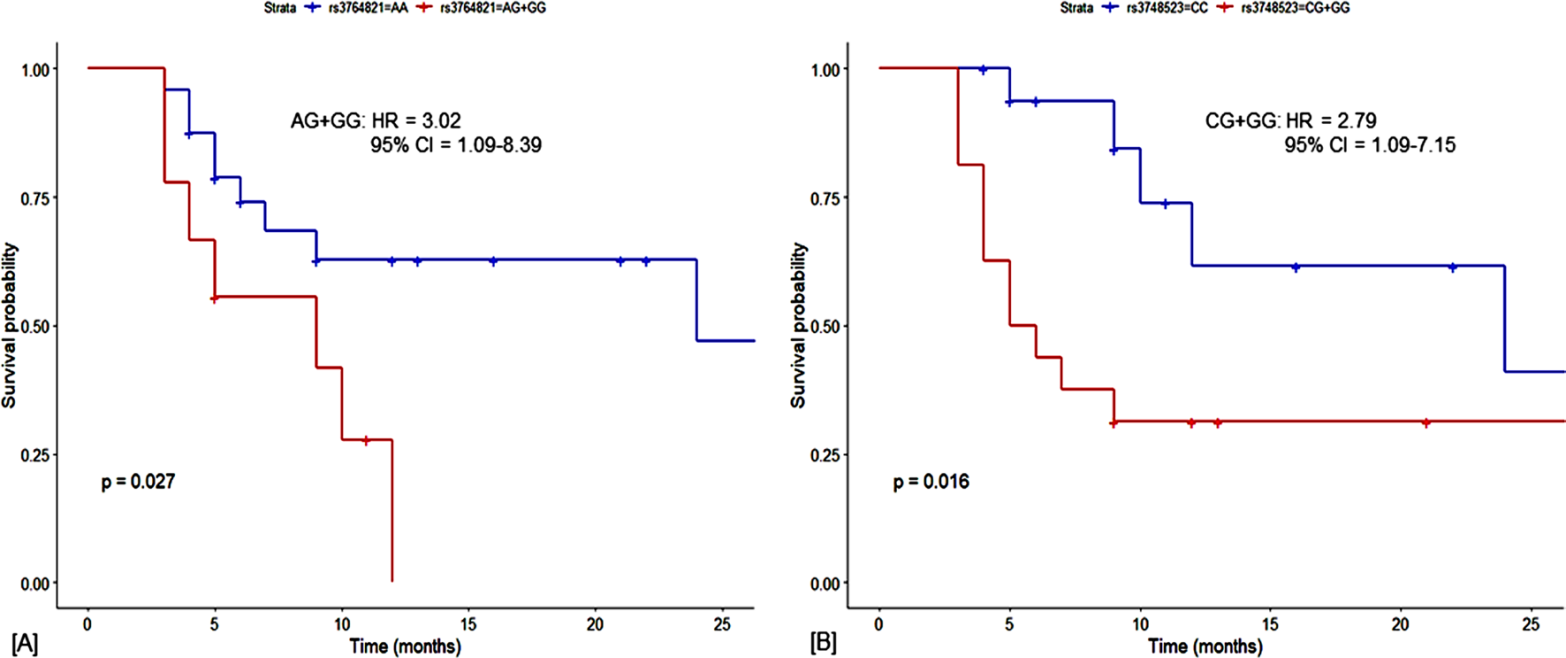
Kaplan–Meier curves depict the association between the polymorphic cis-regulatory variants and overall survival in lung cancer patients treated with different chemotherapy regimens in the eastern Indian population. It shows significantly lower overall survival in lung cancer patients with (**A**) rs3764821 (*ALDH3B1*); treated with gemcitabine-cis/carboplatin in the second regimen and paclitaxel-cis/carboplatin in the first regimen, and (**B**) rs3748523 (*RAD52*); treated with paclitaxel-cis/carboplatin in the first regimen. Significance at log-rank *p* < 0.05*.

For the variant rs3764821 (*ALDH3B1*), lung cancer patient treated with paclitaxel-cis/carboplatin showed a significantly low OS (AA vs. AG + GG: hazard ratio [HR] = 3.62, 95% CI = 1.03–12.71, *p* = *0.044*) in our study population, adjusted for age, sex and pack-years of smoking using a multivariate Cox regression model. Lung cancer patients treated with gemcitabine-cis/carboplatin in the second chemotherapy regimen and paclitaxel-cis/carboplatin (AA vs. AG + GG: hazard ratio [HR] = 4.16, 95% CI = 1.34–12.89, *p* = *0.014*) in the first chemotherapy regimen showed a significant lower *OS* compared to the wild type, adjusted for age, sex and pack-years of smoking using a multivariate Cox regression model (Table 5). Using a KM survival analysis, lung cancer patients treated with gemcitabine-cis/carboplatin in the second chemotherapy regimen and paclitaxel-cis/carboplatin (AA vs. AG + GG: hazard ratio [HR] = 3.02, 95% CI = 1.09–8.39, log-rank *p* = *0.03*) in the first chemotherapy regimen showed a significant lower *OS* compared to the wild type (Fig. 4, Table 5).

In the case of rs3748523 (*RAD52*), lung cancer patients treated with an etoposide-cis/carboplatin regimen showed a higher overall survival (OS) in the study population (CC vs. CG + GG: hazard ratio [HR] = 0.78, 95% CI = 0.10–5.9, *p* = *0.82*) adjusted for age, sex and pack-years of smoking using a multivariate Cox regression model but is not statistically significant. However, lung cancer patients treated with paclitaxel-cis/carboplatin showed significantly lower OS (CC vs. CG + GG: hazard ratio [HR] = 2.79, 95% CI = 1.09–7.15, log-rank *p* = *0.03*) (Fig. 4) in our study population using KM survival analysis. Patients treated with gemcitabine-cis/carboplatin in the second regimen and pemetrexed-cis/carboplatin (CC vs. CG + GG: hazard ratio [HR] = 3.83, 95% CI = 1.39–10.53, *p* = *0.01*) or nanopaclitaxel-cis/carboplatin (CC vs. CG + GG: hazard ratio [HR] = 8.66, 95% CI = 1.33–56.17, *p* = *0.02*) in the first regimen showed a significant lower OS, adjusted for age, sex and pack-years of smoking (Table 5). We also compared the differences in the responses to various drugs in the presence and absence of rs3764821 (*ALDH3B1*) and rs3748523 (*RAD52*) for first-line chemotherapy only. We found that the presence of the variants rs3764821 and rs3748523 showed poor response to Pemetrexed-cis/carboplatin, Etoposide-cis/carboplatin, Paclitaxel-cis/carboplatin and Nanopaclitaxel-cis/carboplatin (Supplementary material Table S26).

Furthermore, we observed a poor response and decreased OS of the lung cancer patients with the variants rs3764821 (*ALDH3B1*) and rs3748523 (*RAD52*) for both first- and second-line chemotherapy. Thus, it reflects the sample population as poor or non-responders to the standard chemotherapy drugs administered to treat advanced lung cancer. In addition, we found rs3748523 (*RAD52*) to decrease OS significantly and showed poor response to first-line Pemetrexed-cis/carboplatin chemotherapy (HR: 3.48, 95% CI = 1.06–11.4, *p* = 0.039*) (Supplementary material Table S27).

## Discussion

Although several studies have implicated many genes and variants with lung carcinogenesis in tobacco smokers, the precise heritable genetic risk signature(s) or prognostic marker(s) is still obscure. Differential gene expression between lung cancer patients with a smoking history and healthy smokers is considered a significant player in lung cancer pathogenesis, particularly for xenobiotic metabolism and DNA repair genes. These two pathways act synergistically to determine the level of carcinogenic load within the lung cells and the capacity to repair DNA damage induced by such carcinogens. We hypothesized that the variants in certain genomic elements regulate such differential gene expression between patients and controls. Therefore, based on this hypothesis, we used the ENCODE data to curate the gene expression-correlated DHS. Such candidate genomic elements could have a cis-regulatory role in gene transcription. The variations within such genomic elements could be the potential modulators of gene expression and need to be characterized to understand the gene regulatory network conferring individual susceptibility to lung carcinogenesis among smokers.

We have designed a workflow to identify, annotate and prioritize such variants within the DHS of genes as risk signatures of lung cancer. We have integrated and interpreted various omics datasets of ENCODE, GTEx, Roadmap Epigenomics, and TCGA datasets through specific web tools to identify, annotate, and prioritize such genetic variants. Out of the 2984 DHS-SNVs in our candidate gene set, only 22 were cis-regulatory in function in lung tissue by integrating and interpreting various omics datasets of ENCODE, GTEx, Roadmap Epigenomics, and TCGA. Transcriptional regulation by genomic elements is tissue-specific^36,65^ and follows a distinctive pattern across the tissues with some conserved elements, while the rest are unique to the cell type. Our study has distinctively identified lung tissue-specific genetic loci responsible for genotype-specific regulation of candidate xenobiotic metabolism and DNA repair gene expression through the analysis of cis-eQTL mapped data. The categorization of rSNPs by the epigenomic signatures into functional gene regulatory chromatin domains provided an insight into the basis of cis-regulatory mechanisms of the genomic elements on their target gene expression. Out of our 22 prioritized cis-eQTLs, we found only 4 significant cis-QTLs in lung cancer from the analysis of TCGA lung cancer datasets harbored in the web tool GEPIA. It further affirmed our workflow’s predictive accuracy and precision as the predicted risk alleles through the pipeline match the reported risk alleles in lung cancer.

Both genome-wide and candidate association studies often reveal unexplained genetic associations with disease/trait, especially for the intronic and intergenic SNPs. We observed nominal associations (*p* < 0.05) of three rSNVs, such as rs35568625 (*MAFG*), rs3760091 (*SULT1A2*), and rs743590 (*SULT1A1*), with lung cancer in 1655 cases and 450,609 controls of all white British origin samples from the UK Biobank GWAS dataset *C34 Malignant neoplasm of bronchus and lung*, hosted by the Gene Atlas webserver (http://geneatlas.roslin.ed.ac.uk/search/), which further strengthened our variant prioritization procedure. Interestingly, the predicted risk alleles of these three rSNVs match the GWAS data, which strengthens our hypothesis and prioritization procedure. However, in an attempt to independently replicate the three rSNVs rs3764821 (*ALDH3B1*), rs3748523 (*RAD52*), and rs5742926 (*PMS1*) from our case–control association study, we failed to find any significant association of the variants with lung cancer in the white British population, which differs from our finding in the east Indian population. The reason for this could be the differences in the population-specific allelic distribution of the variants and the fact that the current study was focused only on smokers. In addition, most of the available lung cancer GWAS datasets represent the Caucasian and East Asian populations, and no such dataset is available on the Indian population.

With the advent of ENCODE and related datasets, scientists are trying to assess if these innocuous loci have any cis-regulatory role on their target genes or are in LD with a cis-regulatory variant that has not been included or filtered out from the specific association study. Detailed analysis indicates that by being in LD, 11 cancer-associated SNPs (5 LD SNPs in lung cancer and other types of cancer, 6 LD SNPs exclusively for different kinds of cancer) might act as surrogates for 8 prioritized rSNVs (3 rSNVs common in lung cancer and non-lung cancer dataset, 1 only in lung cancer dataset and 3 in other cancers). Thus, the finding strengthened our workflow where 5 prioritized cis-regulatory variants are in strong LD with 5 reported lung cancer-associated SNPs. Therefore, it provides transitive evidence of association of the prioritized rSNVs with lung cancer by being in strong LD with reported associations. Again, our revelation of the combination of damaging coding alleles with regulatory risk alleles could result in a significant loss of gene function and thereby have a higher risk modulatory effect in lung carcinogenesis. This could lead to a practical interpretation of the combinatorial role of alleles in a personalized genome approach^40^ for designing therapeutic strategies with precision medicine.

As revealed from our study, the expanded interactome analysis showed strong associations between our prioritized protein-coding genes that provide insight into their probable synergistic influence in mitigating tobacco smoke-induced damage. Interaction of critical proteins, such as TP53, has been found to interact with the NFE2L2 pathway indicating a vital relationship between the xenobiotic metabolism and cellular transformation pathways that paved the way for future investigations on cytoprotection and tumorigenesis. The cross-talk of the detoxification and DNA repair pathway with cytoskeletal remodeling, metastasis, apoptosis, and cell cycle regulatory pathways provides an insight into the carcinogen-induced gene regulatory mechanisms in lung carcinogenesis among smokers.

The prioritized genes have diverse functions related to the metabolism of tobacco smoke components and repairing oxidative DNA lesions induced by smoke carcinogens that form the basis of risk allele determination. We have summarized the probable impact of the risk alleles on the gene function contributing toward lung carcinogenesis among smokers (Supplementary Material, Table S28).

Earlier genome-wide association studies (GWAS) have shown rs10849605 of *RAD52* significantly associated with an increased risk of lung cancer^66^. Our data found a significant association of rs3748523 of RAD52 with an increased risk of lung cancer, implicating collinearity in the studies for gene function in lung cancer. This is the first report on the regulatory polymorphism of *ALDH3B1,* significantly altering lung cancer risk by regulating the detoxification potential of the enzyme. However, the *PMS1* gene shows an association with lung cancer^63^ in Caucasians. However, the lack of association of rs5742926 of *PMS1* in our study could be attributed to the sample size due to low minor allele frequency in the eastern Indian population. It is worth mentioning that rs3748523 of the *RAD52* gene is associated with lung cancer in low smokers of a young age. This indicates the potential role of the variant in reducing the expression of the DNA repair gene, conferring the early risk of lung cancer in individuals with low to medium smoking intensity. Earlier reports have indicated an association between tobacco and betel quid chewing and lung cancer^67,68^.

Interestingly, rs3764821 of *ALDH3B1* and rs3748523 of *RAD52* were associated with lung cancer in tobacco and betel quid chewers. The risk genotype of both polymorphisms would cause ineffective metabolism of the xenobiotics from tobacco and betel quid and sub-optimal DNA repair of DNA damages caused by the constant xenobiotic load. Thus, the combinatorial inheritance of risk alleles of the SNPs would confer a higher risk of developing lung cancer, and stratifying the genotypes based on tumor subtypes and TNM staging improved risk assessment. Prediction of the risk for specific tumor subtypes and cancer stages leads to the design of targeted early detection and prevention strategies. Moreover, identifying histotype-associated SNPs may define the mechanism underlying the unknown origins of morphological variations and contribute to a personalized treatment approach for subtype-specific lung cancer cases^69^.

In the present study, we have also evaluated the role of two lung cancer-associated regulatory polymorphic variants in the survival of lung cancer patients treated with platinum-based chemotherapy. None of the variants showed any improvement in the overall survival of patients post-treatment with standard platinum-based chemotherapy. However, the risk alleles of the polymorphic variants were found to significantly lower the overall survival of lung cancer patients post platinum-based chemotherapy, adjusted for covariates like age, sex, and pack-years of smoking. We found a significant reduction in OS in patients with the risk allele of rs3764821 (*ALDH3B1*), treated with gemcitabine-cis/carboplatin as a second line of treatment after paclitaxel-cis/carboplatin. This could be due to the lower expression of *ALDH3B1* that causes an inadequate response to platinum-based chemotherapy leading to higher systemic toxicity and increased mortality among the advanced-stage (IIIB and IV) NSCLC patients in our sample population. To the best of our knowledge, this is the first study that reports the role of cis-regulatory polymorphic variants in modulating the overall survival in eastern India lung cancer patients post-treatment with standard chemotherapy. Therefore, *ALDH3B1* and *RAD52* play a pivotal role in tobacco smoke-induced lung carcinogenesis and platinum-based standard chemotherapy, which could be critical prognostic markers of the disease and predictors of chemotherapy responses. Aldehyde dehydrogenase, including *ALDH3B1,* is involved in the detoxification and clearance of chemotherapeutic drugs, leading to chemotherapy resistance^70,71^. Similarly, the RAD52 is a DNA-binding protein that repairs single-strand DNA breaks introduced by the genotoxic compounds in tobacco smoke^72,73^. A lower expression of both genes would imply impaired detoxification of tobacco smoke metabolites and the repair of DNA damage introduced by the same tobacco smoke metabolites, influencing overall survival and the efficacy of chemotherapy regimens with different drug combinations.

A limitation of this approach is the difficulty of getting the necessary sample sizes, given the relative rarity of many such histological subtypes or the lack of proper clinical records. However, our data mining approach with prior knowledge of the disease etiology helped prioritize the most relevant SNVs for replication, even in a small sample size. Furthermore, due to the lack of high-resolution HiC and ChIA-PET datasets on lung tissue, a more detailed analysis of the physical interaction of cis-elements, particularly promoter-enhancer/repressors, could not be done.

The co-occurrence of risk alleles and estimation of unweighted genetic risk scores (uGRS) of 22 prioritized rSNPs provided insight into individual and population-specific tobacco-dependent lung cancer. The preponderance of the risk alleles stratified by sub-populations of 1000 Genome data predicted the Gambians in Western Gambia (GWD) to be at risk while the Americans of African Ancestry in South West USA (ASW) to be at least risk. Traditionally, insufficient epidemiological studies on lung cancer incidences in the African population led to inconclusive risk assessment a priori. A recent development in maintaining nationwide cancer registries in different countries of the continent increased the coverage to 13% of the population, which is a deviation from the earlier notion of Africans being the most protected population against tobacco smoking-related lung cancer. The increase in lung cancer incidences throughout the African continent, mainly in West Gambia and the sub-Saharan region, could be attributed to the increase in tobacco smoking and the aging of the predisposed population^74^. However, on stratification based on the larger geospatial population of 1000 Genome data, Europeans were at high risk of tobacco smoke-dependent lung carcinogenesis, substantiated by epidemiological reports^74^. Lung cancer rates showed a 20-fold variation stratified by region, which predominantly reflects the decrease in patterns of tobacco exposure, including intensity and duration of smoking, type of cigarettes, and degree of inhalation in the developed world. A diminution in smoking prevalence among men caused a decline in lung cancer rates in several high-income countries where smoking was first established, including the United Kingdom, Finland, the United States, the Netherlands, Australia, New Zealand, Singapore, Germany, and Uruguay. Recent reports in 26 European countries revealed a decline in age-standardized (35–64 years) incidences of lung cancer, with Bulgaria as an exception^55^. Therefore, being susceptible to tobacco-dependent lung cancer, the Europeans probably managed to reduce the disease load by changing their lifestyle habits^75^. All of these showcase the importance of this work towards identifying risk populations and designing effective tobacco control policies to reduce lung cancer incidences. Epidemiological reports^76,77^ corroborate our finding that Latinos/Non-white Hispanics are at the lowest risk of tobacco smoke-dependent lung cancer among all the other populations of the 1000 Genome data, followed by the Africans. Despite high smoking rates, lung cancer incidences are pretty low in the Central and South American Latinos/ Non-white Hispanic population^76,77^. In future studies, we would try to corroborate the weighted genetic risk score of the variants with the epidemiological data of lung cancer from the global lung cancer datasets.

The study has implied a pathway-based approach to identify 22 cis-regulatory variants of 14 genes (XMGs and DRGs) through integrating and interpreting various freely available omics data. The cross-validation of the statistical association of the identified rSNVs with lung cancer by their LD-SNPs and the precise match of the risk alleles of the cis-eQTLs in lung cancer to normal tissue shows the success of our prioritization pipeline. The case–control replication following the in silico prioritization provides population-specific risk markers of lung carcinogenesis. Incorporating more genes of critical lung cancer regulatory pathways would enable us to construct a comprehensive, personalized genomic map of individuals across different populations for assessing their lung cancer risk profiles to design personalized therapy based on precision medicine and formulating effective tobacco control policies and genetic counseling for the containment of the disease. We opine that the method followed in this study for identifying cis-regulatory risk markers of lung carcinogenesis among smokers could be implied to other complex diseases or traits.

## Supplementary Information

Supplementary Figures.

Supplementary Tables.

## Data Availability

All data generated or analyzed during this study are included in this article and its supplementary information files. The additional raw input data files will be available from the corresponding authors upon request.

## Code availability

The R scripts/codes used for the analysis will be available from the corresponding author upon request.
