# Supplementary material for: Identifying polymorphic cis-regulatory variants as risk markers for lung carcinogenesis and chemotherapy responses in tobacco smokers from eastern India
Source: Sci Rep. 2023 Mar 10;13:4019. doi: 10.1038/s41598-023-30962-9 (PMC10006236; doi:10.1038/s41598-023-30962-9)
Supplement: Supplementary file 1 — Supplementary Figures. [file 41598_2023_30962_MOESM1_ESM.docx]

**Supplementary Figures**

**
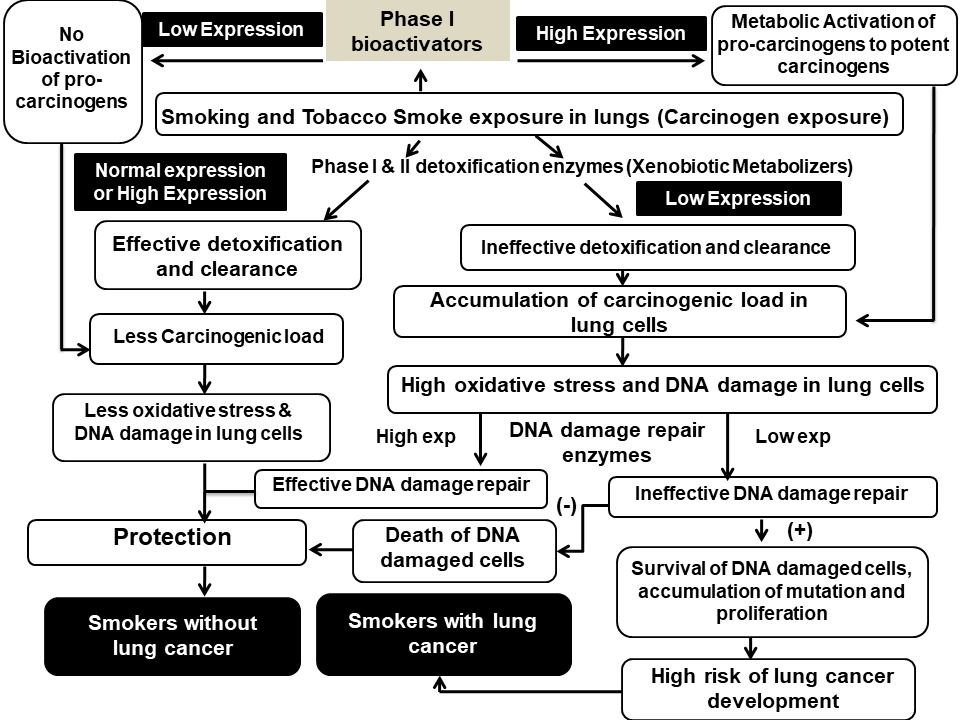
**

**Figure S1. Pathway of tobacco smoke metabolism and damage repair involved in tobacco smoke-induced lung carcinogenesis.** The impaired function of xenobiotic metabolism and DNA repair genes leads to carcinogenic potentiation.

**
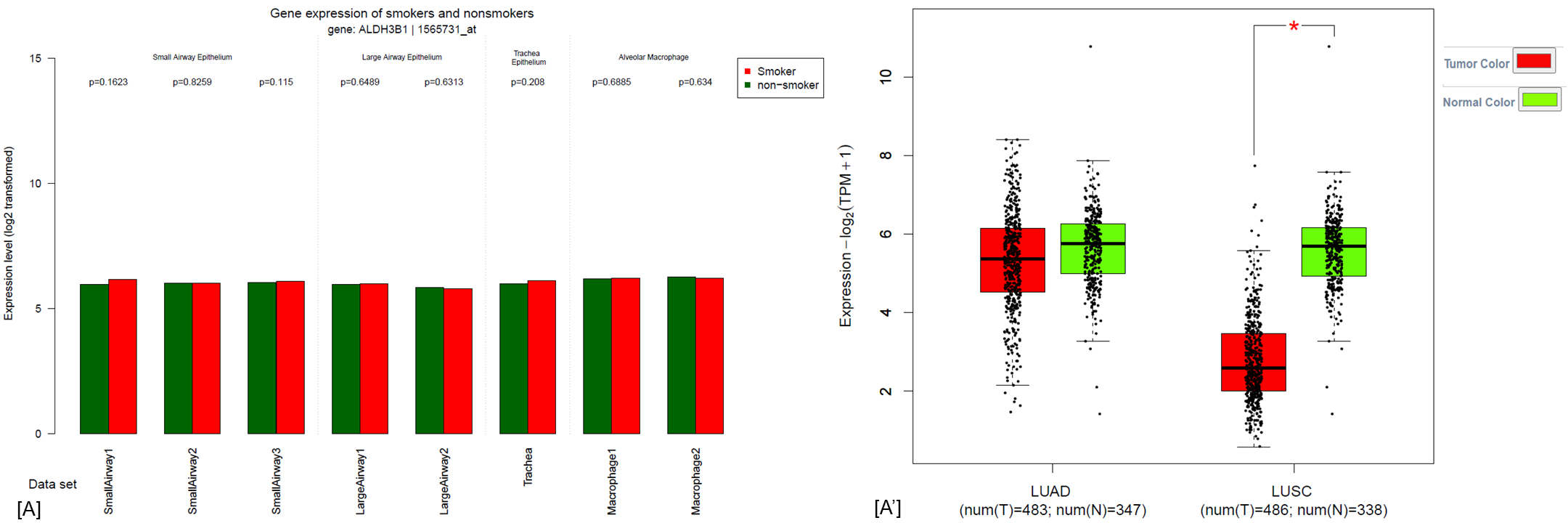
**

**
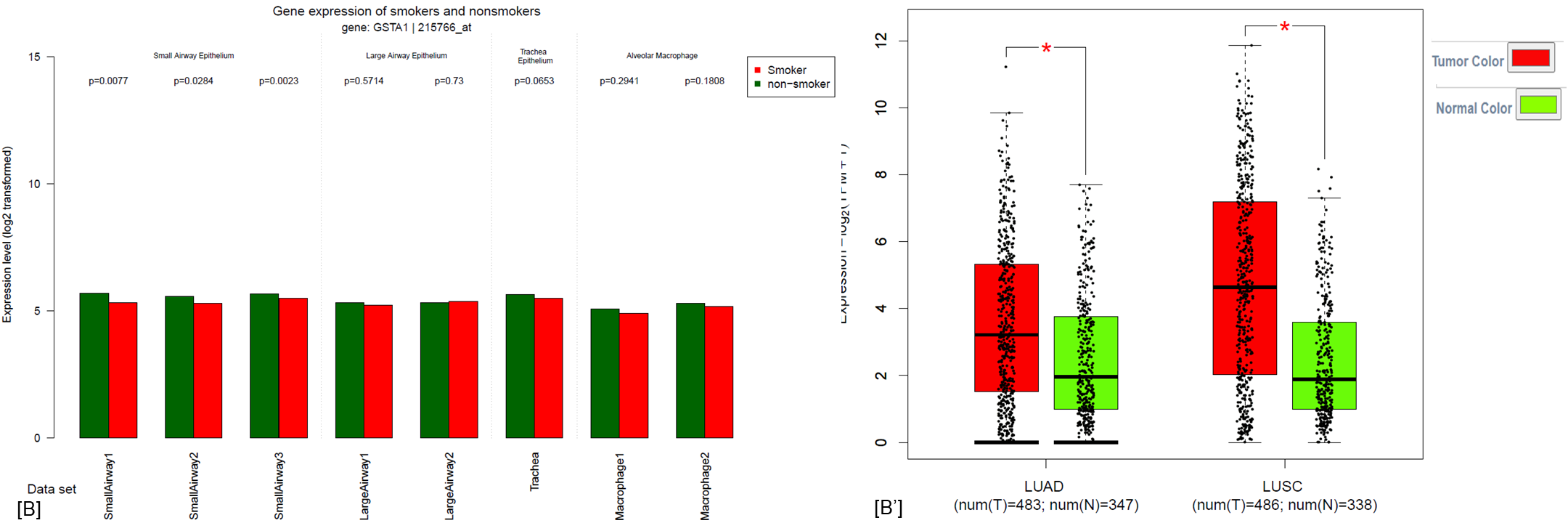
**

**
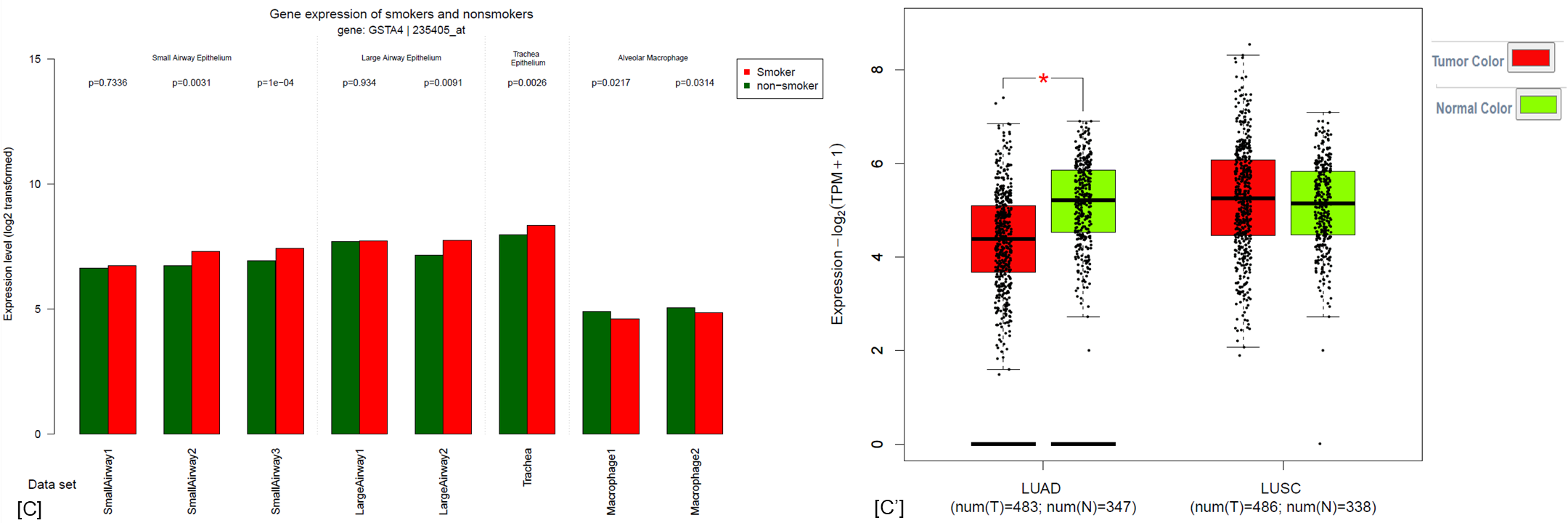
**

**
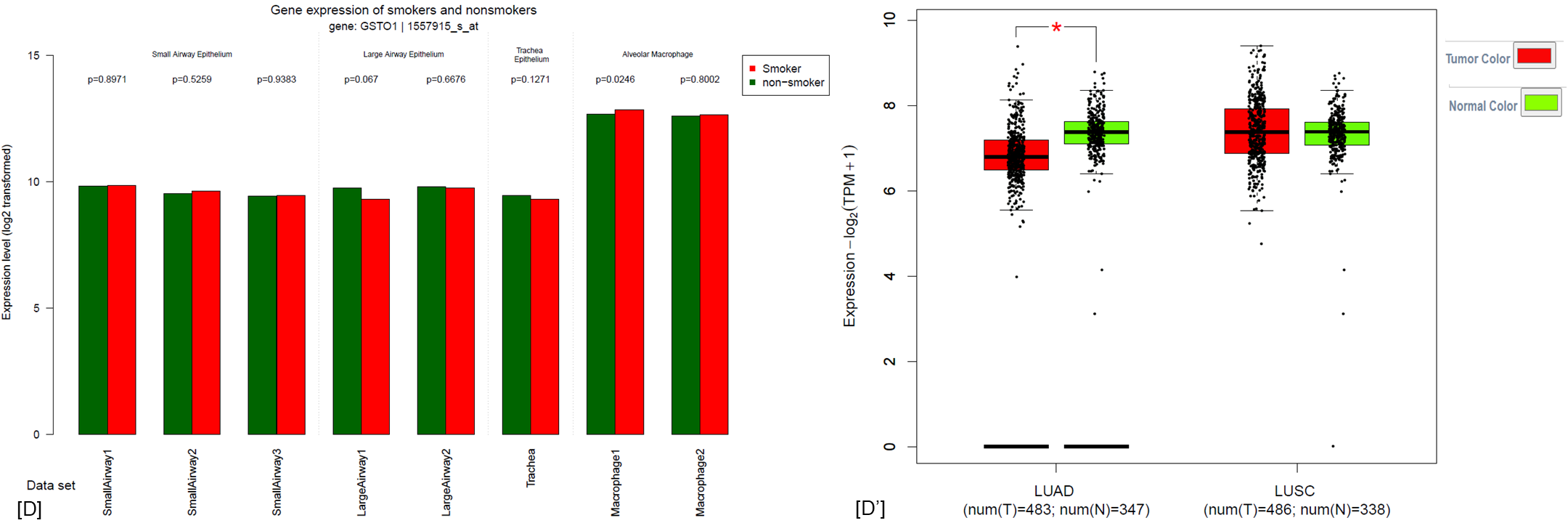
**

**
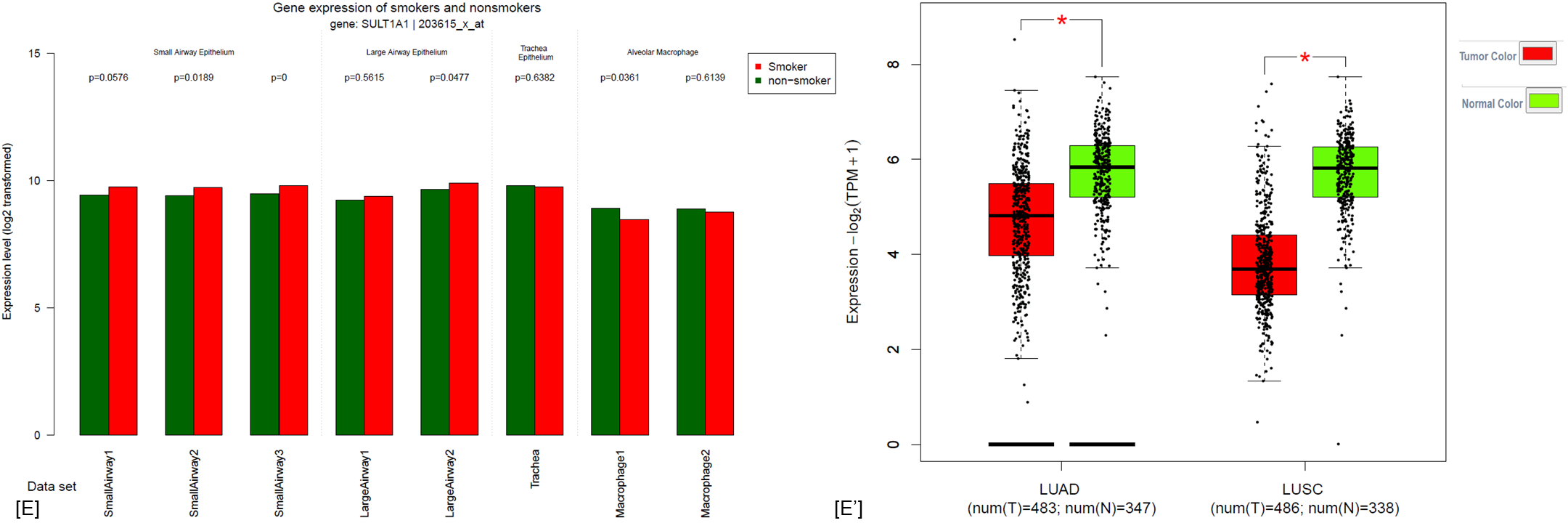
**

**
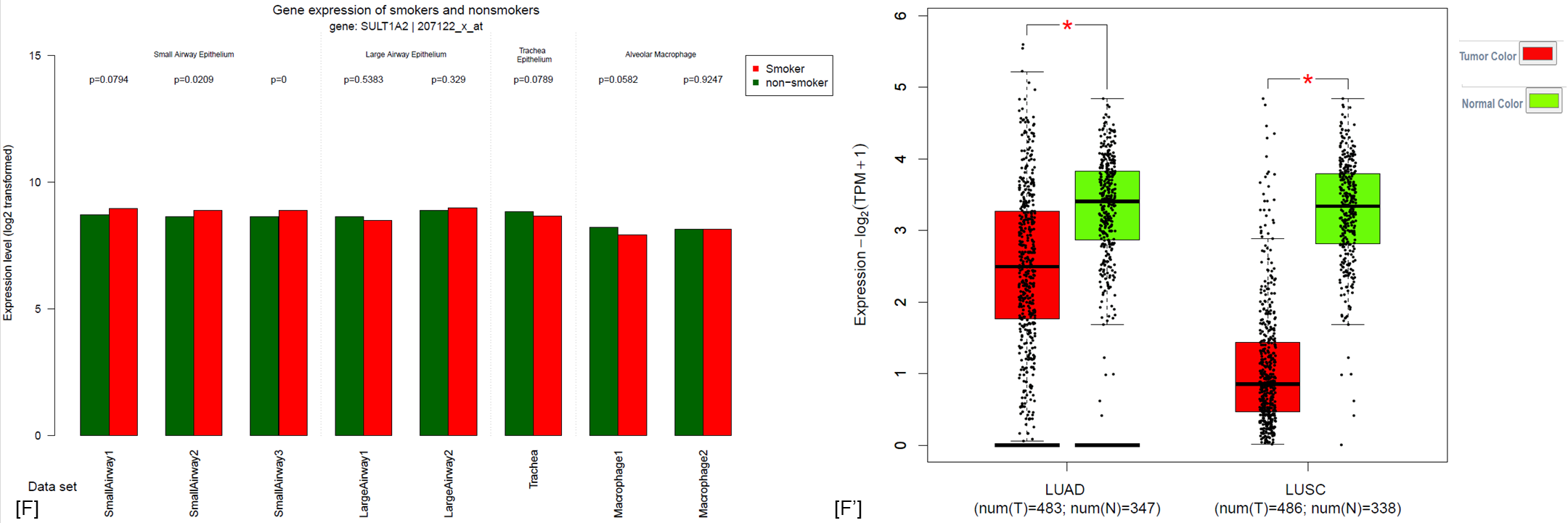
**

**
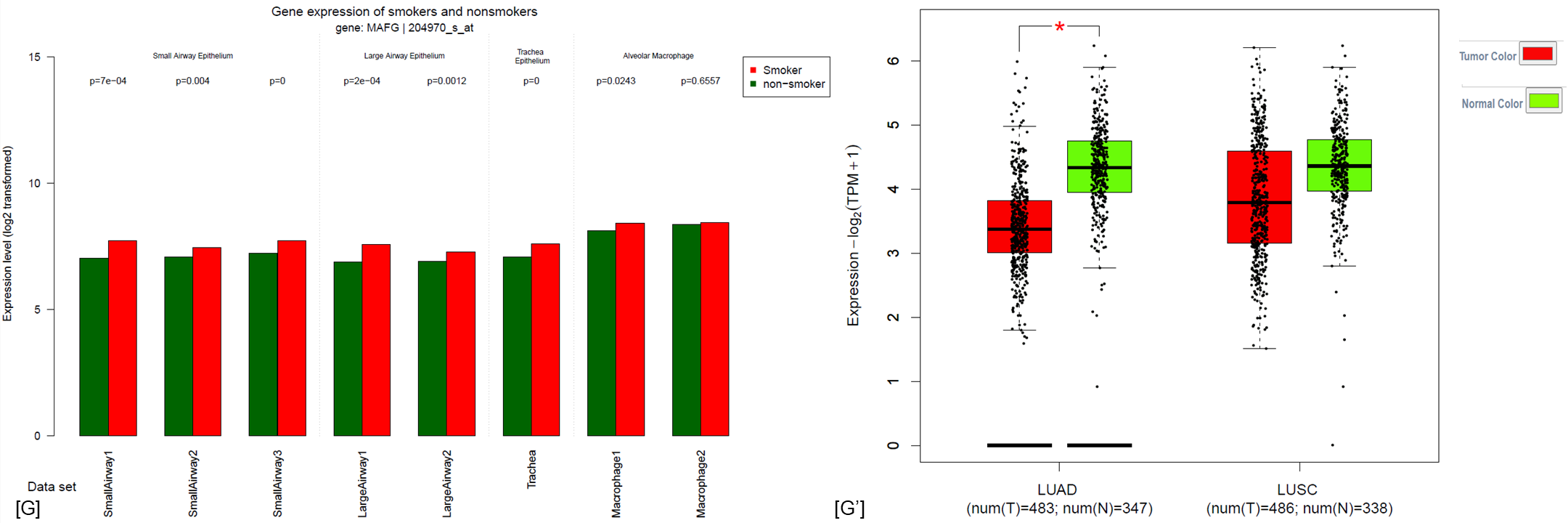
**

**
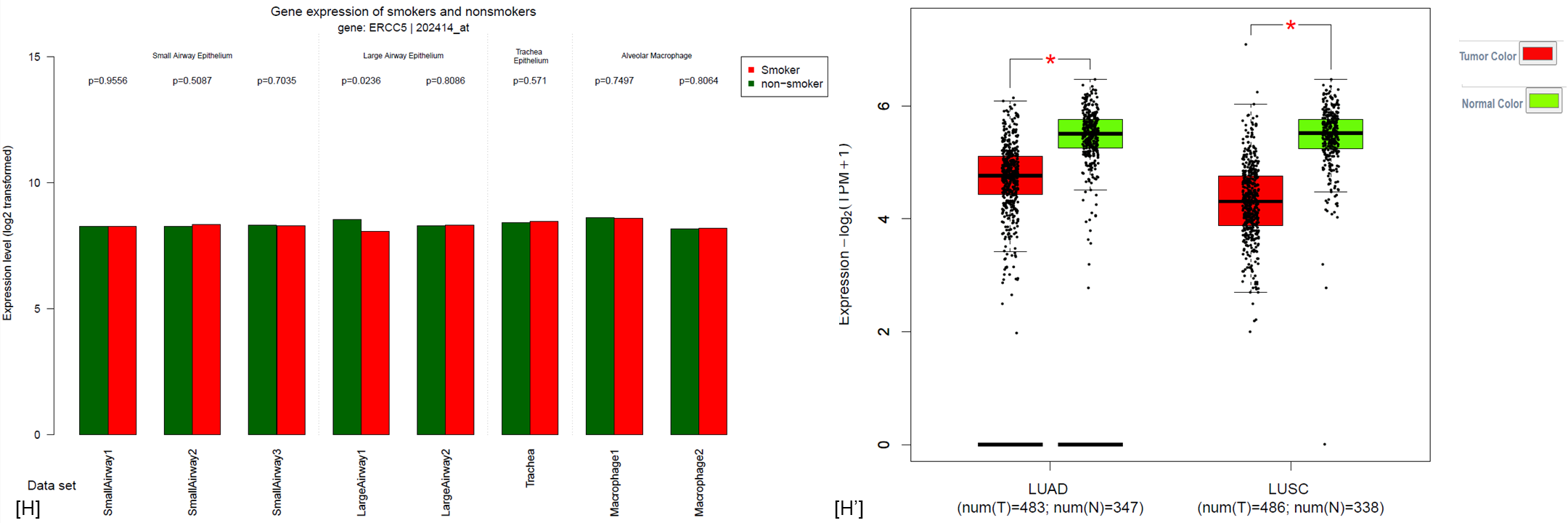
**

**
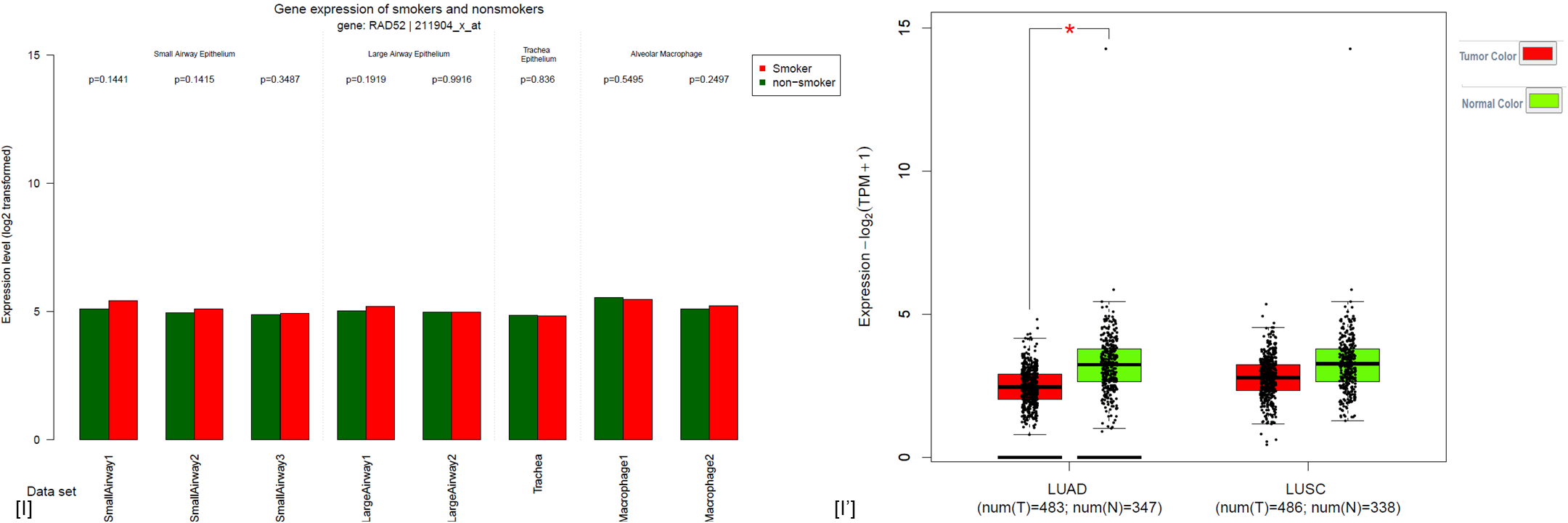
**

**
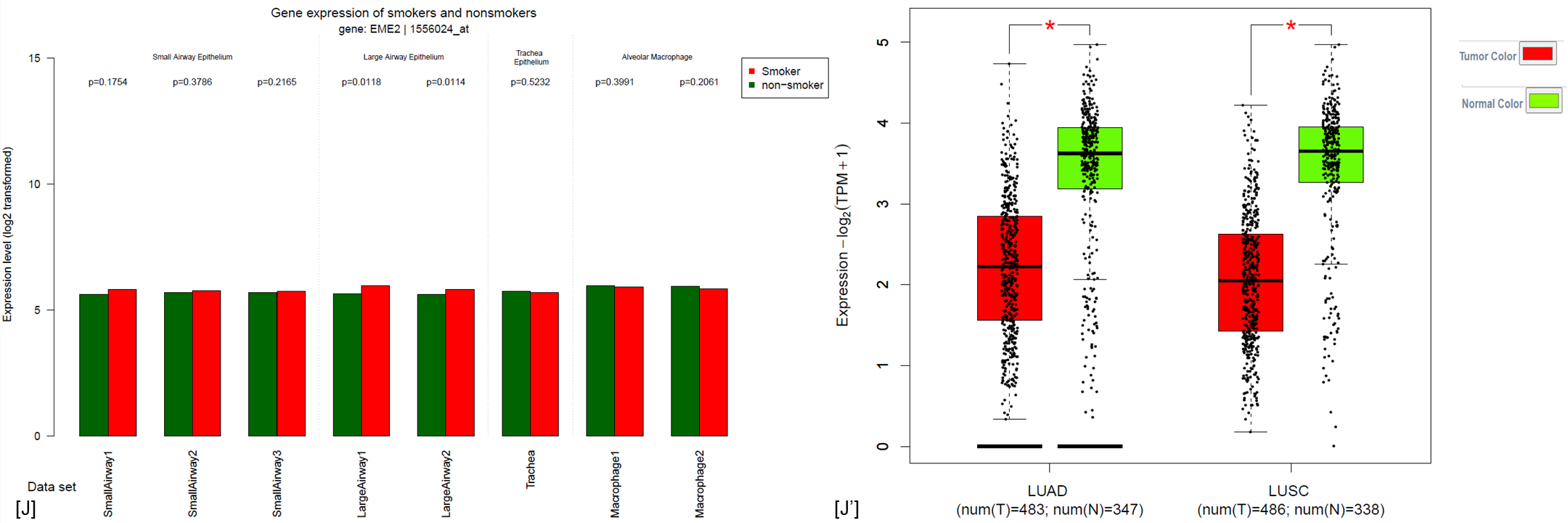
**

**
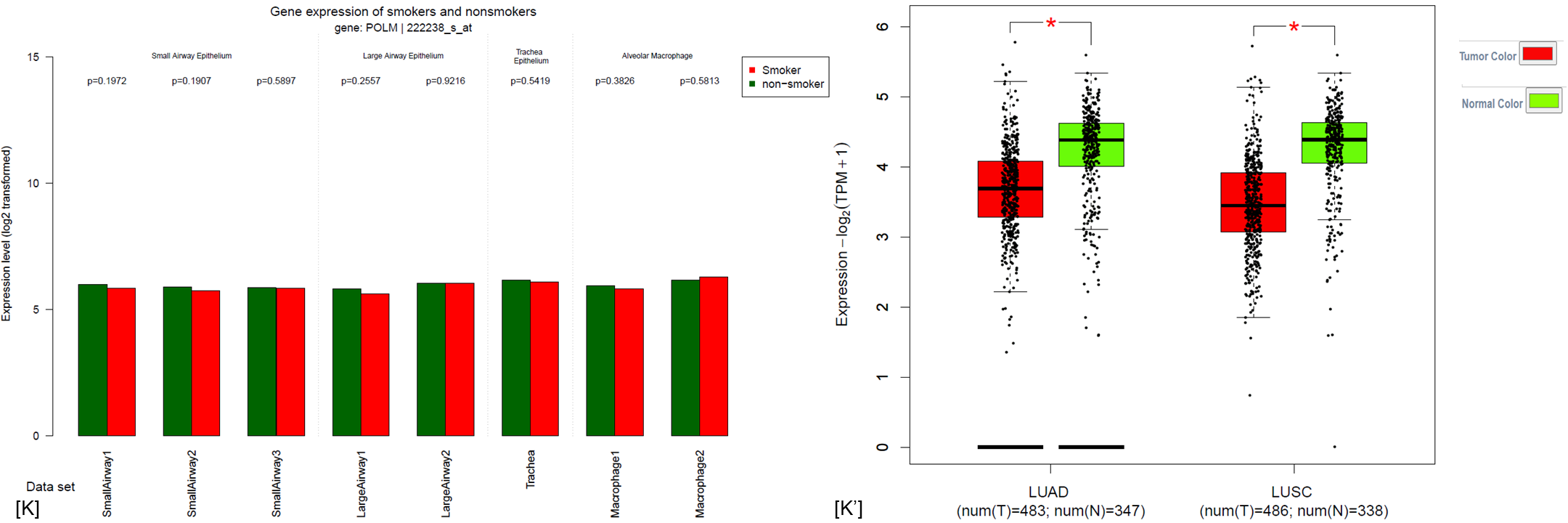
**

**
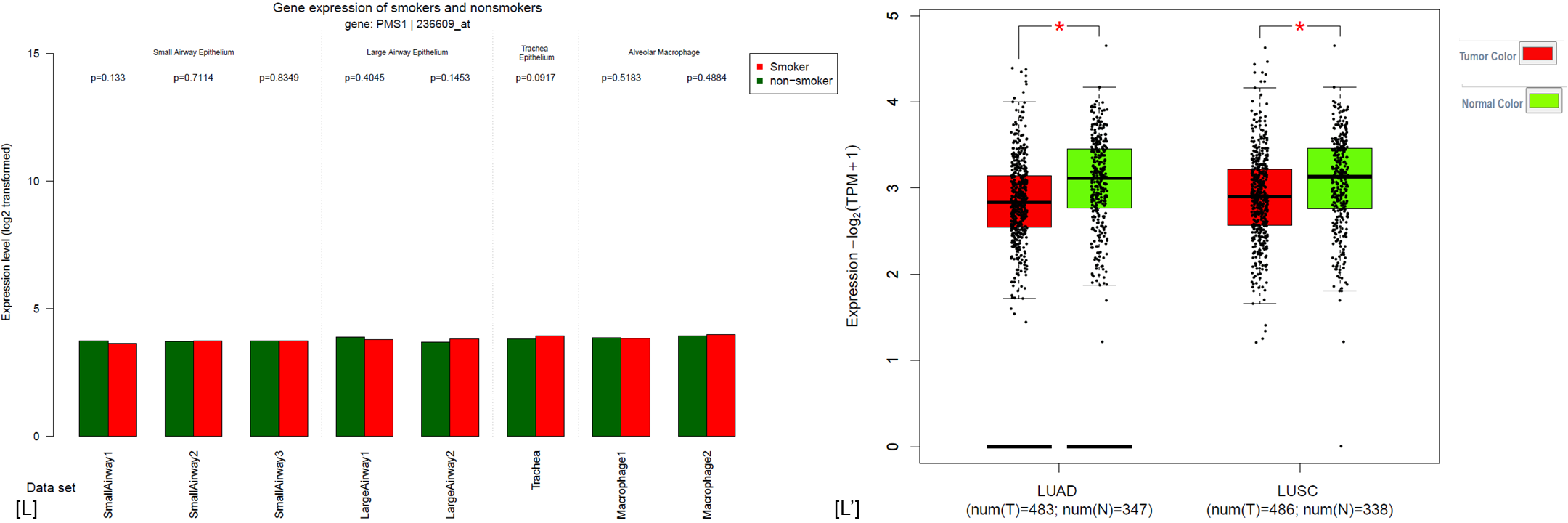
**

**
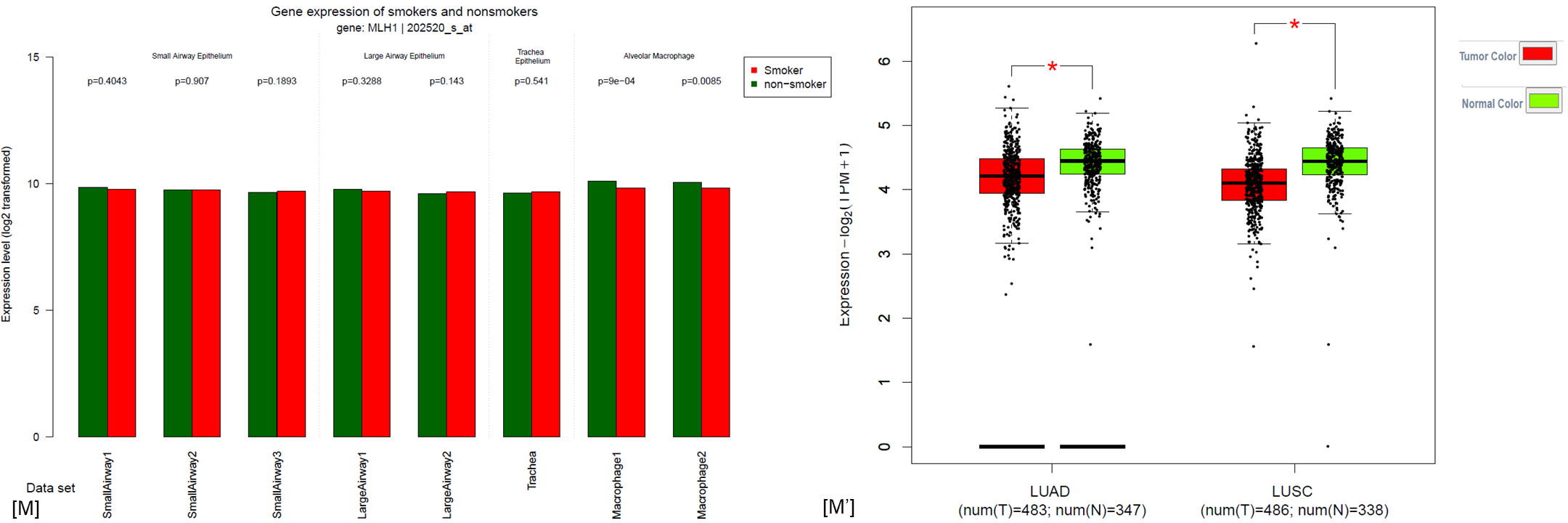
**

**Figure S2. Graphical representation of the differential expression of genes between HS vs HNS (A-M) obtained from the SEGEL database and between SLC vs HS (A’-M’) from GEPIA webserver.** *Level of significance p< 0.05*, 0.01**, 0.001****.















**Figure S3. A box plot representation depicts genotype to tissue expression of significant cis-eQTLs (*p* < 0.05*) in healthy lung tissue (A-E).** Data was obtained from the GTEx portal. ‘N’ represents the number of individuals.


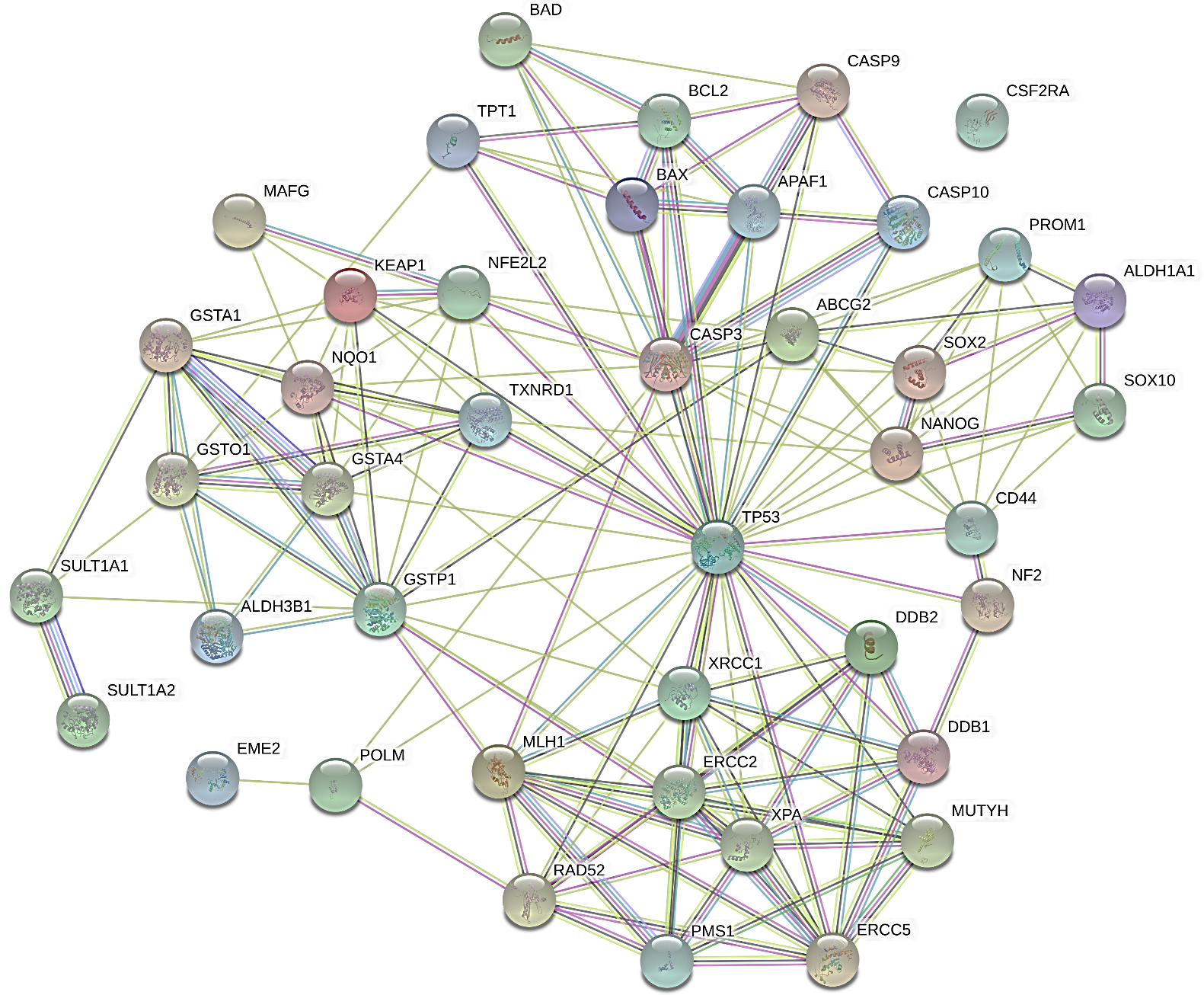


**Figure S4. A Protein-Protein Interactome for the final set of prioritized genes in STRING v10.5.** The interaction was expanded to view more interactors and identify potential candidate protein-coding genes.


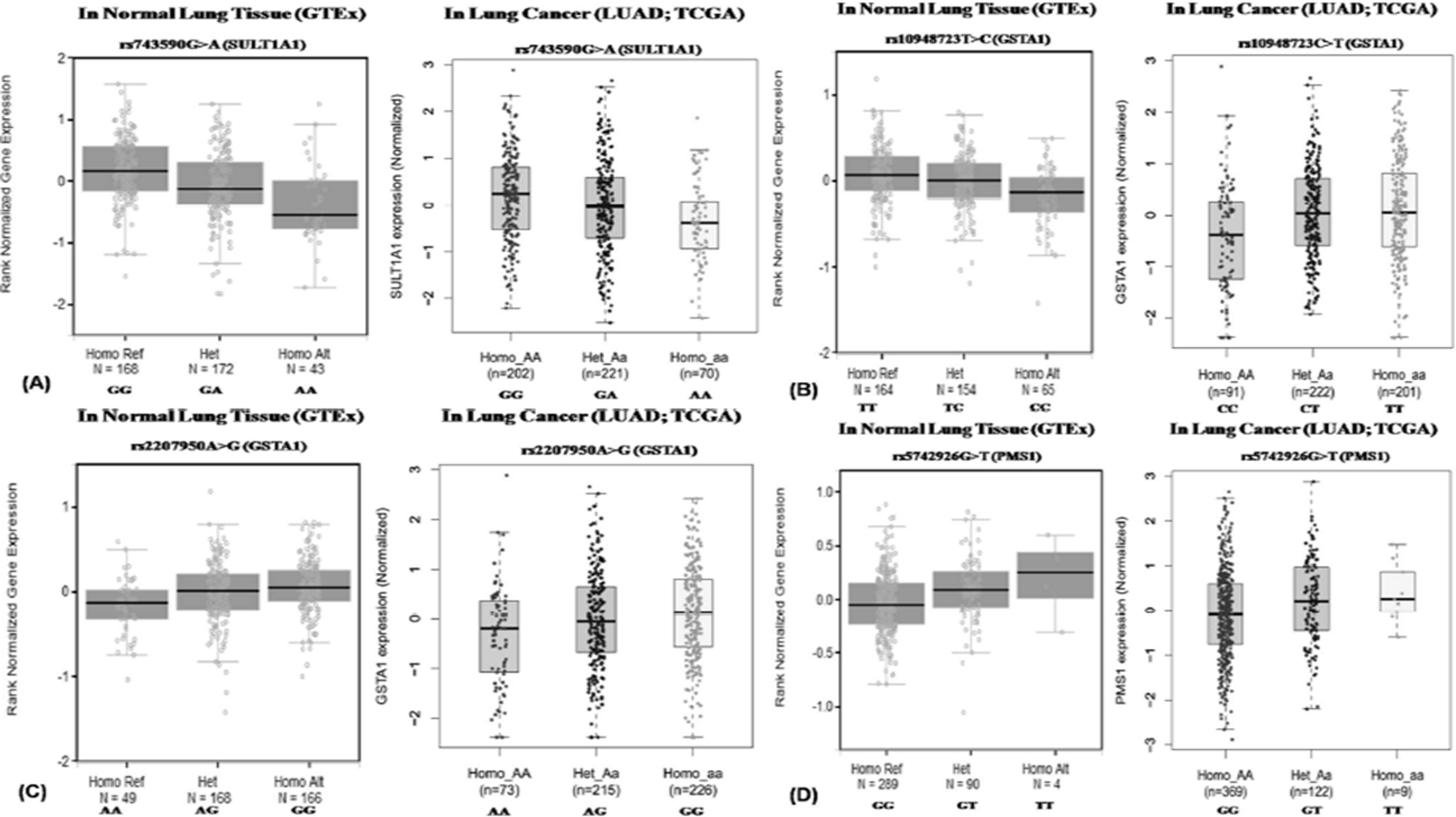


**Figure S5. A comparative box plot representation depicting the genotype-specific expression of 4 cis-eQTL rSNPs from 3 genes in lung cancer and healthy individuals**. Data for lung cancer, both lung adenocarcinoma, and lung squamous cell carcinoma, were taken from TCGA in the PancanQTL web server. For healthy lung tissue, the data was analyzed through the GTEx portal. ‘N’ represents the number of individuals. FDR-corrected *P*<0.05* is considered significant.
